# Efficient synthesis of phenylene-ethynylene rods and their use as rigid spacers in divalent inhibitors

**DOI:** 10.3762/bjoc.9.25

**Published:** 2013-01-31

**Authors:** Francesca Pertici, Norbert Varga, Arnoud van Duijn, Matias Rey-Carrizo, Anna Bernardi, Roland J Pieters

**Affiliations:** 1 Department of Medicinal Chemistry and Chemical Biology, Utrecht Institute for Pharmaceutical Sciences, Utrecht University, P.O. Box 80082, 3508 TB Utrecht, The Netherlands; 2 Dipartimento di Chimica, Universita’ degli Studi di Milano, via Golgi 19, 20133 Milano, Italy

**Keywords:** multivalent carbohydrates, LecA inhibition, phenylene ethynylene, rigid spacers, Sonogashira reaction

## Abstract

The synthesis of phenylene-ethynylene rods and their use as rigid spacers is described. Alternation of a Sonogashira reaction and silyl group cleavage was used to obtain rigid spacers with even and odd numbers of phenylene units. Preliminary applications of these rods in divalent systems are shown. Inhibition studies with *Pseudomonas Aeruginosa* lectin LecA showed that the rigid spacer proved greatly beneficial for the inhibitory potency.

## Introduction

Linker or spacer molecules have a wide range of applications in many areas of chemistry as bridging molecules between separate functional units. Spacers are often flexible but depending on the nature of the application, efforts have been made to make rigid linkages between functional units. Examples of these have been reported in areas such as nanoelectronics and nanooptics [1], surfactants [2–4], photoelectrochemical detection [5], catalysis [6], glycosylation reactions [7], and carbohydrate–protein interactions [8]. Many different strategies and molecule types have been used depending on the desired geometry, such as ring formation [9], carbohydrate–triazole conjugation [10], aryl–alkyne linked structures [11–15] and the use of DNA as a rigid bridge between silver nanoparticles and quantum dots [5]. Among the rigid linking units the phenylene-ethynylene unit has seen considerable interest in sensor [16] and molecular-electronics applications [17]. This is due to the specific fluorescent, conducting and electrochemical properties that the conjugated system confers to the molecule [18]. In the study of carbohydrate–protein interactions, it is now well known that making a system multivalent increases the binding or inhibitory potency of ligands, whose monovalent counterparts would otherwise be too weak to have biological relevance [19–22]. The spacer is an important factor in the design of an effective multivalent ligand [23]. While most systems reported thus far contain flexible spacers, there is a major untapped potential for systems based on rigid spacers, even though some flexibility may be needed to overcome design imperfections [10].

**Figure 1:**
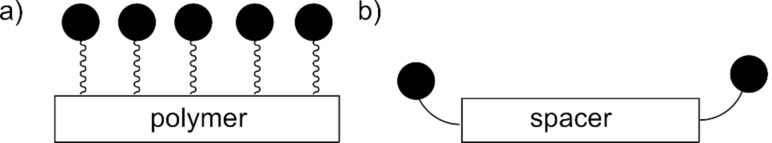
Schematic depiction of (a) a rigid phenylene-ethynylene polymer core with ligands attached via flexible chains, and (b) a divalent ligand using phenylene-ethynylene as a rigid spacer connecting the ligands.

We herein describe the synthesis of rigid spacers of various lengths based on phenylene-ethynylene units and their incorporation into divalent ligands. For design purposes this system has the advantage that due to the high rigidity and linearity it is easy to predict their length, as was recently shown by EPR measurements [24]. The solubility of rigid hydrophobic spacers in an aqueous environment is notoriously poor, and therefore PEG attachments have been employed. Such PEG units were also incorporated in glycopolymers based on the phenylene-ethynylene repeating units by Seeberger and co-workers (see schematically in Figure 1a), who used them for the detection of bacteria [25]. The fluorescent properties of the polymer allowed a fast detection of the *E. coli* bacteria. Brewer et al. reported a divalent inhibitor with a short rigid spacer containing just a single phenylene-ethynylene [26]. The inhibition shown by this compound was disappointing since it was less potent than its flexible divalent counterpart. There are no other examples in the literature that show inhibition studies of lectins using divalent ligands directly connected by a rigid spacer based on phenylene-ethynylene units (Figure 1b).

**Figure 2:**
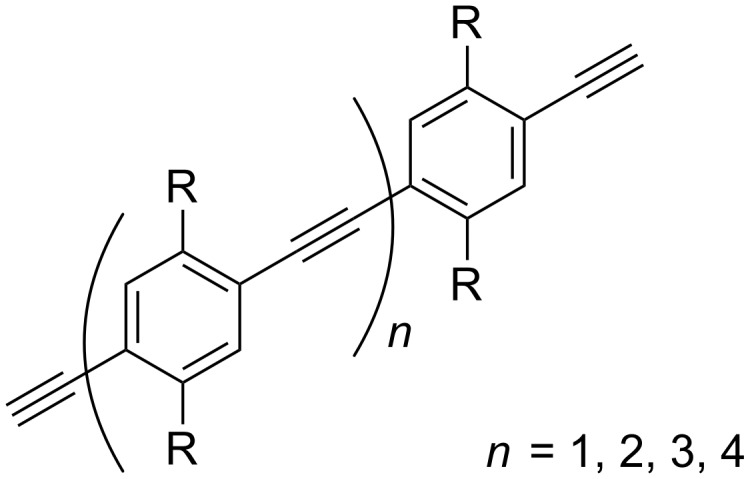
Generic structure of spacers containing an even (*n* = 1, 3) and an odd (*n* = 2, 4) number of units.

In order to systematically study the effects of spacer lengths on the binding potencies of divalent ligands, having access to a series of spacers of well-defined lengths was imperative. We here report the synthesis of a series of spacers based on phenylene-ethynylene building blocks (Figure 2), with distinct syntheses for the compounds containing an even and an odd number of aromatic rings. One of the spacers was incorporated into the structure of a divalent galactoside ligand and was used to inhibit the virulence-linked lectin LecA of *Pseudomonas aeruginosa* [27–28].

## Results and Discussion

### Synthetic strategies

**Figure 3:**
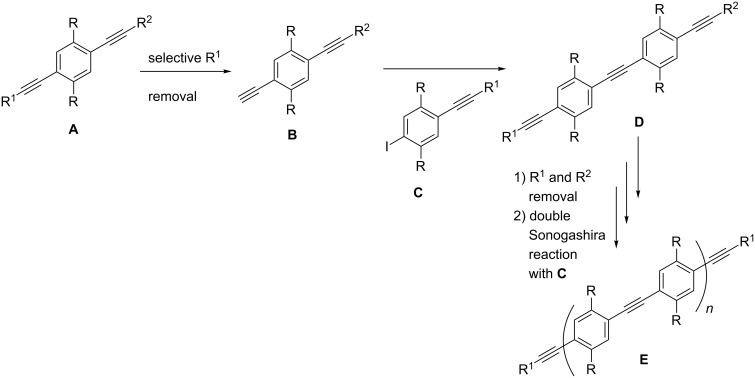
Synthetic strategy for rigid spacers with an even number of units.

Depending on the number of units in the spacer, two different routes can be applied. The pathway followed to obtain rods containing an even number of units is shown in Figure 3. The R group on the ring is used to increase the solubility of the system. The strategy relies on orthogonal protecting groups R^1^ and R^2^ of structure **A**, to enable the selective deprotection needed to make **B**. Its free alkyne moiety can undergo a Sonogashira reaction with **C** to give **D**. At this point the removal of the protecting group R^1^ and R^2^ can be performed, to either couple the ligands or elongate the system by a double Sonogashira reaction.

**Figure 4:**
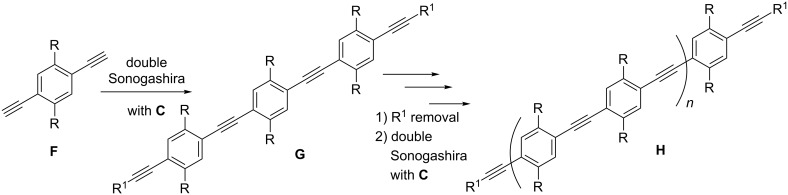
Synthetic strategy for rigid spacers with an odd number of units.

The strategy to prepare spacers containing an odd number of units is shown in Figure 4. The strategy is more straightforward since it does not require any orthogonal deprotection step. Starting with **F**, a double Sonogashira reaction with **C** should yield the three-unit system **G**. Removal of the protecting groups R^1^ can be performed, to either couple the ligands or elongate the system by a double Sonogashira reaction.

### Synthesis of the building blocks

**Scheme 1:**
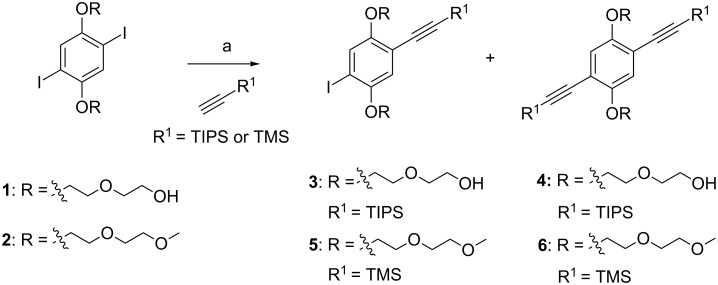
Synthesis of building blocks; (a) from **1**, Pd(PPh_3_)_4_ , CuI, PPh_3_, TEA, toluene, 50 °C, 5 h, 31% for **3** and 50% for **4**; from **2**, PdCl_2_(PPh_3_)_2_ , CuI, TEA, THF, microwave, 60 °C, 20 min, 35% for **5** and 53% for **6**.

The building blocks were prepared as shown in Scheme 1. In the general structures shown in Figure 3 and Figure 4 the R group is used to increase the solubility. For this purpose diethylene glycol was used as a side chain, which terminated as a free hydroxy group for **1** and a methoxy group for **2**. Silyl groups were used as selective protective groups for the alkyne moiety. Monoalkyne **3** and bisalkyne **4** were made from **1** [29] by a Sonogashira reaction with TIPS-acetylene in 31% and 50%, respectively. Similarly, **5** and **6** were obtained from **2**, in agreement with a recent literature report [16]. The use of the microwave reactor allowed a shorter reaction time (20 min at 60 °C) than the one reported in the literature (24 h at 40 °C for **6** and 24 h at 10 °C for **5**).

### Synthesis of a two-unit spacer

**Scheme 2:**
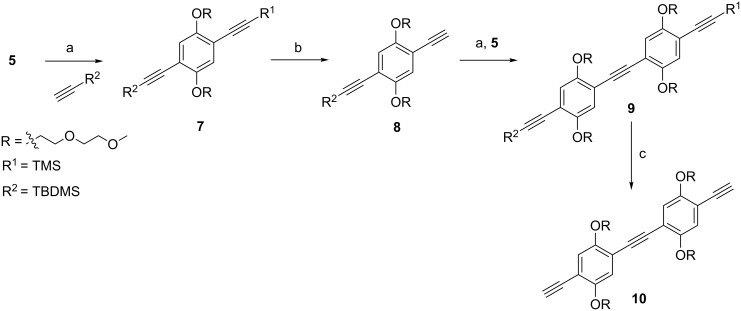
Synthesis of a two-unit spacer. (a) PdCl_2_(PPh_3_)_2_, CuI, TEA, THF, microwave, 60 °C, 20 min, 81% for **7**, 79% for **9**; (b) K_2_CO_3_, MeOH/CH_2_Cl_2_, 45 min, 78%; (c) TBAF, THF, 84%.

The strategy of Figure 3 was applied to the synthesis of the two-unit spacer. In order to obtain our orthogonally protected intermediate **7**, mono-iodo compound **5** was coupled with *tert*-butyl(ethynyl)dimethylsilane (TBDMS-acetylene, Scheme 2). From **7** the more labile TMS group was removed by using K**_2_**CO**_3_** to give **8**. This compound was coupled to **5** by a Sonogashira reaction to give the protected two-unit spacer **9**. Removal of its silyl protecting groups with TBAF yielded the desired two-unit spacer **10.** A slightly different strategy (see Supporting Information, Scheme S1) was used starting from the mono-iodo derivative **3**, because partial silyl migration to the free hydroxy groups was observed while attempting mono-desilylation.

### Synthesis of three-unit spacers

**Scheme 3:**
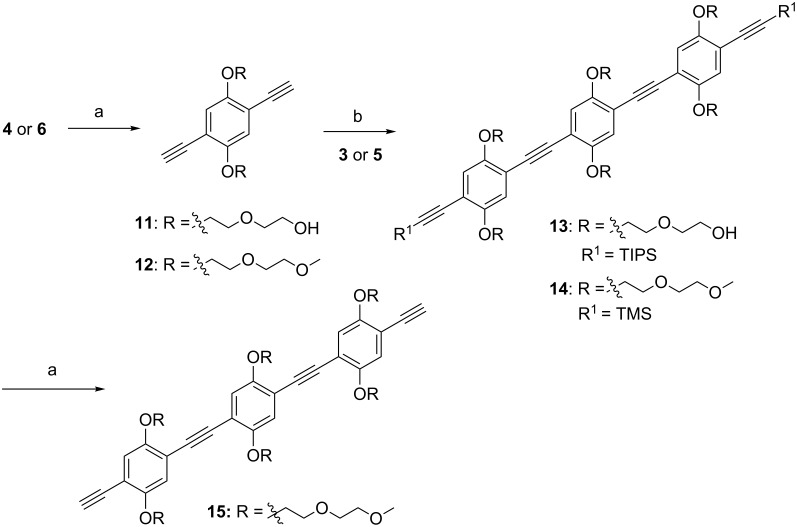
Synthesis of three-unit spacers. (a) from **4**: TBAF, THF, 62%; from **6** and **14**: K_2_CO_3_, MeOH/CH_2_Cl_2_, 45 min, 85–88%; (b) from **11**: Pd(PPh_3_)_4_, CuI, PPh_3_, TEA, toluene, 50 °C, 14 h, 50%; from **12**: PdCl_2_(PPh_3_)_2_, CuI, TEA, THF, microwave, 60 °C, 20 min, 75%.

To obtain the three-unit spacer, the odd strategy (Figure 4) was applied starting with compound **F**. In order to make **F**, the silyl protecting groups of both **4** and **6** were removed (Scheme 3). For **4** the TIPS groups were removed with an excess of TBAF to give **11**. Compound **6**, which bears the TMS group, was treated with K_2_CO_3_ to afford **12**. Both **11** and **12** were elongated in a double Sonogashira reaction with **3** and **5**, respectively. Of the products, **14** was deprotected by using K_2_CO_3_ to yield the three-unit spacer **15**, according to a recent literature report [16].

### Synthesis of a four-unit spacer

**Scheme 4:**
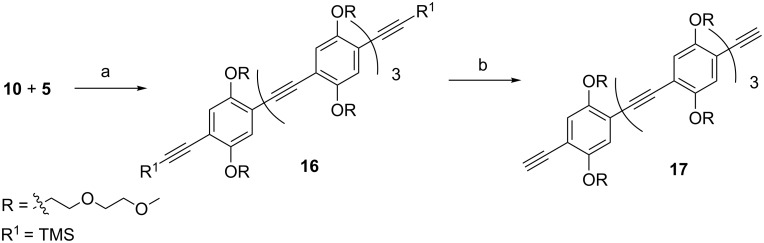
Synthesis of a four-unit spacer. (a) PdCl_2_(PPh_3_)_2_, CuI, TEA, THF, microwave, 60 °C, 20 min, 73%; (b) K_2_CO_3_, MeOH/CH_2_Cl_2_, 45 min, 76%.

The four-unit spacer was synthesized starting from the two-unit spacer **10** and the iodo compound **5** through a double Sonogashira reaction to give **16** (Scheme 4). Deprotection of the two alkyne moieties with K_2_CO_3_ afforded the four-unit spacer **17**.

### Synthesis of a five-unit spacer

**Scheme 5:**
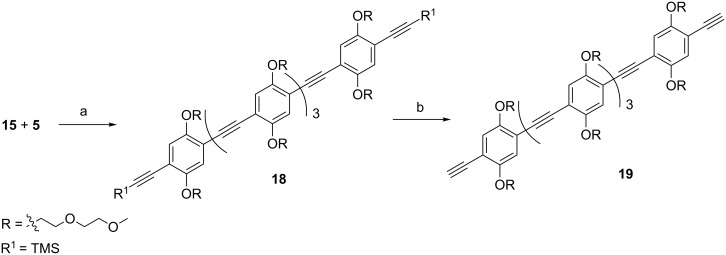
Synthesis of a five-unit spacer. (a) PdCl_2_(PPh_3_)_2_, CuI, TEA, THF, microwave, 60 °C, 20 min, 70%; (b) K_2_CO_3_, MeOH/CH_2_Cl_2_, 45 min, 75%.

The synthesis of the five-unit spacer started with the elongation of the three-unit spacer **15** through a double Sonogashira reaction with iodo compound **5** to give **18** (Scheme 5). The five-unit spacer **19** was obtained after deprotection of the alkyne moieties with K_2_CO_3_.

### Preliminary application

**Scheme 6:**
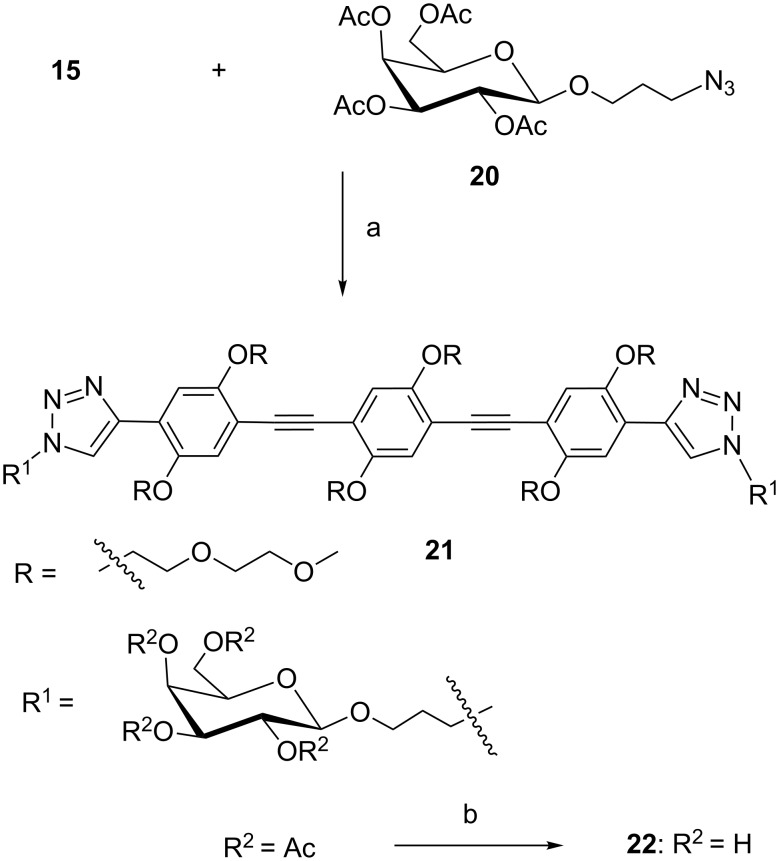
Synthesis of divalent ligand **22**. (a) CuSO_4_·5H_2_O, Na-ascorbate, DMF/H_2_O, microwave, 80 °C, 40 min, 85%; (b) NaOMe, MeOH, 41%.

As part of our program on bacterial adhesion inhibition by multivalent carbohydrates, the bacterial lectin LecA, a virulence factor of the problematic pathogen *Pseudomonas aeruginosa* is a target of interest [30–31]. This tetrameric lectin binds galactosides and the shortest distance between two binding sites is around 26 Å [20,28]. We previously noted that the use of a rigid spacer with some flexibility in the aglycon chain connecting the galactose ligands, led to more potent inhibition [10]. Since the distance to cover is ca. 26 Å, the best match in the phenylene-ethynylene series is the three-unit spacer **15.** Just the spacer, without the aglycon linking moiety measures around 22 Å. Coupling of the galactose ligand and the flexible aglycon part should result in a promising compound. CuAAC of **15** with the ligand **20** was performed as shown in Scheme 6 to give **21**. The acetyl protecting groups were removed by using NaOMe in MeOH, yielding final product **22**.

**Scheme 7:**
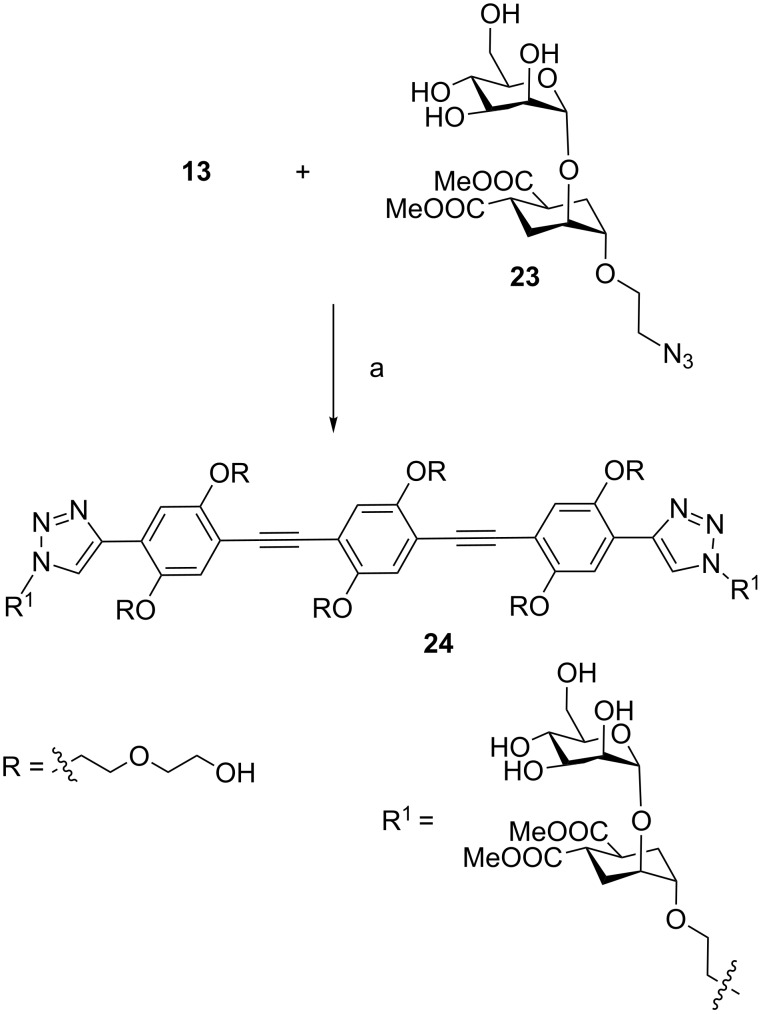
Synthesis of divalent ligand **24**. (a) **13**, TBAF in THF, rt, 1 h, then H_2_O, TBTA, CuSO_4_·5H_2_O, Na-ascorbate, **23**, rt, 18 h, 76%.

Compound **13**, bearing hydroxy-terminated diethylene glycol chains, has the potential of solubilizing in water constructs bearing active spearheads less hydrophilic than simple sugars. We have been developing pseudo-disaccharide molecules such as **23** [32] (Scheme 7) as mimics of mannose disaccharides for the interaction with DC-SIGN and other C-lectins [33–35]. This molecule and its derivatives [36] contain lipophilic moieties that generally increase their affinity for the target proteins, but can create solubility problems. Desilylation of **13** (TBAF, THF, Scheme 7) followed by in situ CuAAC with the pseudo-disaccharide **23** led to the divalent ligand **24**, which was found to be fully soluble in water, at least up to millimolar concentrations. Compound **24** is a mimic for DC-SIGN inhibition and its bioactivity will be tested elsewhere.

### Inhibition studies

**Figure 5:**
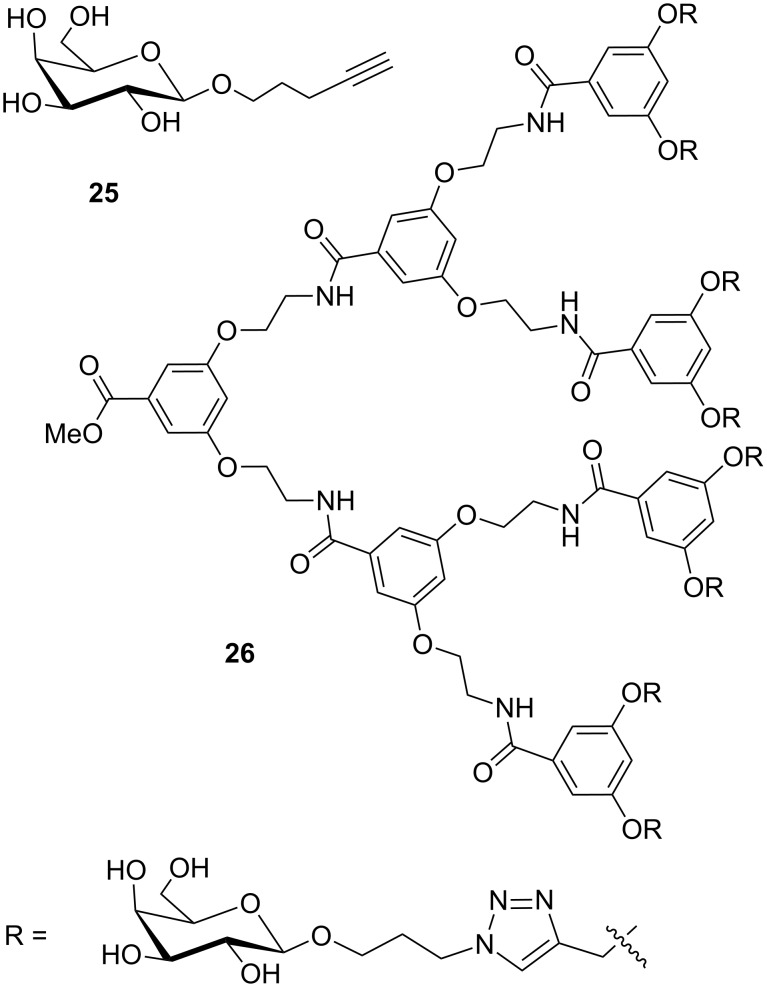
Previously tested compounds.

The inhibitory potency of **22** for LecA was studied in an ELISA type assay by using a glycochip as the solid phase [10]. In this assay an IC_50_ value of 0.9 μM was determined (Table 1). This compared favorably with the monovalent reference compound **25** (Figure 5), which exhibited an IC_50_ of 120 μM [10]. Similarly, the divalent compound **22** was a more potent inhibitor than the higher valency compound **26**, especially when expressed as the relative potency per sugar, which is 11 for the tetravalent **26** [37–38] and 67 for **22**.

**Table 1:** Inhibitory potency of mono, di- and tetravalent galactosides on LecA binding^a^.

Compound	Valency	IC_50_/μM	Relative Potency (per sugar)

**25**	1	120	1
**26**	4	2.7	44 (11)
**22**	2	0.90	133 (67)

^a^FITC-labeled LecA, 20 μg mL^−1^ binding to a galactoside functionalized surface.

## Conclusion

In this work a strategy for the synthesis of rigid spacers or rods of different length based on phenylene-ethynylene units was developed. On the phenyl ring, two versions of a solubilizing diethylene glycol moiety were used, one terminating in a hydroxy and one terminating in a methoxy group. The hydroxy version led to some migration of the silyl protecting group upon attempted monodeprotection, and alternative strategies had to be devised for the synthesis of the two-unit spacer. Compounds **17** and **19**, containing four and five phenylene-ethynylene units, respectively, can still be further elongated, depending on the need of the project, thus enabling the preparation of long spacers with a well-defined number of monomeric units. The CuAAC of the three unit spacer **15** with a galactose ligand gave the divalent ligand **21** in good yield. After deprotection this compound was used to inhibit the lectin LecA from *Pseudomonas aeruginosa*. A major potency increase was seen with the divalent structure based on the phenylene-ethynylene spacer, with an IC_50_ of 0.9 μM, i.e., a 133-fold potency increase over a monovalent reference compound, clearly showing the potential for spacers of this nature.

It was also shown that the three-unit spacer **13** could be used in a one-pot desilylation and CuAAC reaction to give **24**, which was found to be fully soluble in water, despite the more lipophilic nature of the active ligand. This finding paves the way for the synthesis and evaluation of polyvalent glycomimetics with regularly spaced cores to be used for Man-specific C-lectin inhibition assays. With access to structural data of target lectins, the design of multivalent inhibitors can be performed on a customized level, where the selection of the proper spacer is based on the information about the binding site, such as its density, orientation and position. Furthermore, the rigidity of the rods described above can contribute to overcoming the entropic penalty of flexible multivalent scaffolds, thus improving the overall activity of the ligands.

## Supporting Information

File 1 Synthetic procedures and spectral data.
