# Supplementary material for: Efficient synthesis of phenylene-ethynylene rods and their use as rigid spacers in divalent inhibitors
Source: Beilstein J Org Chem. 2013 Jan 31;9:215–22. doi: 10.3762/bjoc.9.25 (PMC3566787; doi:10.3762/bjoc.9.25)
Supplement: File 1 — Synthetic procedures and spectral data. [file Beilstein_J_Org_Chem-09-215-s001.pdf]

Supporting Information
for

Efficient synthesis of phenylene-ethynylene rods and their use as rigid spacers in divalent inhibitors

Francesca Pertici[1], Norbert Varga[2], Arnoud van Duijn[1], Matias Rey-Carrizo[1], Anna Bernardi[2] and Roland J. Pieters[1,]*

Address: [1]Department of Medicinal Chemistry and Chemical Biology. Utrecht Institute for Pharmaceutical Sciences, Utrecht University, P.O. Box 80082, 3508 TB Utrecht, The Netherlands and [2]Dipartimento di Chimica, Universita' degli Studi di Milano, via Golgi 19, 20133 Milano, Italy

Email: Roland J. Pieters* - R.J.Pieters@uu.nl.

* Corresponding author

Synthetic procedures and spectral data

General: Unless stated otherwise, chemicals were obtain from commercial sources and were used without further purification. Compounds **1** [1], **2** [2] and **20** [3] were prepared according to the literature procedures. Solvents were purchased from Biosolve (Valkenswaard, The Netherlands). All moisture-sensitive reactions were performed under a nitrogen atmosphere. Anhydrous THF was dried over Na/benzophenone and freshly distilled prior to use. All the other solvents were dried over molecular sieves 4 Å or 3 Å**.** TLC was performed on Merck precoated Silica 60 plates. Spots were visualized by UV light and 10% H_2SO_4 in MeOH. Microwave reactions were carried out in a Biotage microwave Initiator (Uppsala, Sweden). The microwave power was limited by temperature control once the desired temperature was reached. Sealed vessels of 2–5 mL and 10–20 mL were used. Analytical HPLC runs were performed on a Shimadzu automated HPLC system with a reversed-phase column (Alltech, C8, 90 M, 5 mm, 250 L, 4.6 mm, Deerfield, IL, USA) that was equipped with an evaporative light-scattering detector (PLELS 1000, Polymer Laboratories, Amherst, MA, USA) and a UV–vis detector operating at 220 nm and 250 nm. Preparative HPLC runs were performed on an Applied Biosystems workstation. Elution was effected by using a linear gradient of 5% MeCN/0.1% TFA in H_2O to 5% H_2O/0.1% TFA in MeCN. ^{1}H NMR (300 MHz) and ^{13}C (75.5 MHz) were performed on a Varian G-300 spectrometer. HSQC and HMBC NMR (500 MHz) were performed with a VARIAN INOVA-500. ^{1}H and ^{13}C spectra for compounds **3**,

4, **13** and **24** were recorded at 400 MHz on a Bruker AVANCE-400 and 300 MHz on Bruker DPX-300 instrument. The numbering for protons in the NMR characterization are shown on the molecules, due to the symmetry of the molecule it does not correspond to the numbering used for nomenclature. Electrospray mass experiments were performed in a Shimadzu LCMS QP-8000. High-resolution mass spectrometry (HRMS) analysis was performed using an Applied Biosystems 4700 MALDI TOF/TOF instrument for compound **22**. Mass spectra of **3**, **4** and **13** were obtained with a ThermoFisherLCQapparatus (ESI ionization) or Apex II ICR FTMS (ESI ionization-HRMS) for compound **24**.

LecA inhibiton assay: The lectin LecA was obtained from Sigma-Aldrich and it was FITC labeled according to the procedure of Sigma-Aldrich [4]. Microarray experiments were performed by using PamChip arrays run on a PamStation12 instrument (Pam-Gene B.V., ’s-Hertogenbosch, the Netherlands). Data were obtained by real-time imaging of the fluorescence signal by a CCD camera. Images were analyzed by using BioNavigator software (Pam-Gene). Each array slide contains spots in duplicate. The fluorescence intensities were expressed in arbitrary units and the relative intensities were the average of the two duplicate spots. Aliquots of a solution of FITC-labeled LecA (20 μg mL^{-1}) in HEPES/PBS buffer (10 mM HEPES, 100 mM NaCl, 0.1% BSA. pH 7.4), containing different concentrations of the inhibitors were incubated for 1 h at rt and subsequently added to the glycodendrimer chip. The binding process was monitored for 2 h and the end values of the fluorescence detection were taken for the determination of the IC50 by using Prism 5 (Graphpad Software, Inc).

1,4-bis[2-(2-hydroxyethoxy)ethoxy]-2-[tris(1-methylethyl)silyl]ethynyl)-5-iodobenzene (3) and 1,4-bis[2-(2-hydroxyethoxy)ethoxy]-2,5-bis[tris(1-methylethyl)silyl]ethynyl)benzene (4)

1 (50 mg, 0.093 mmol, 1 equiv), $Pd(PPh_3)_4$ (4 mg, 0.003 mmol, 0.04 equiv), CuI (6.5 mg, 0.003 mmol, 0.04 equiv), and PPh_3 (2.3 mg, 0.009 mmol, 0.1 equiv) were placed into the reaction flask and dried under vacuum, then the reagents were dissolved in toluene (0.6 mL) and finally ethynyltriisopropylsilane (TIPS-acetylene, 25.4 mg, 0.139 mmol, 1.5 equiv), and Et_3N (0.2 mL) were added under nitrogen. The reaction was heated to 50 °C for 5 h. The reaction was diluted with ethyl acetate, filtered through a silica pad and the filtrate was concentrated under reduced pressure. The resulting crude was purified by flash chromatography (silica, hexane with gradient of ethyl acetate from 0 to 90%) to afford 30.3 mg (31%) of product **3** and 16.3 mg (50%) of product **4**.

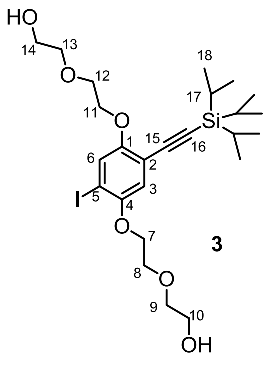

3

^{1}H NMR (400 MHz, $CDCl_3$) δ 7.27 (1H, s, H_6), 6.87 (1H, s, H_3), 4.15–4.05 (4H, m, H_7, H_{11}), 3.93–3.78 (4H, m, H_8, H_{12}), 3.77–3.58 (8H, m, H_9, H_{10}, H_{13}, H_{14}), 1.16–0.86 (3H, m, H_{17}), 1.10–1.12 (18H, m, H_{18}); ^{13}C NMR (100 MHz, $CDCl_3$) δ 155.0 (C_1); 152.0 (C_4); 124.3 (C_6); 117.6 (C_3); 114.4 (C_2); 102.4 (C_{15}); 96.7 (C_{16}); 87.8 (C_5); 72.8, 72.8 (C_{13}, C_9); 70.1, 69.8, 69.7,

69.5 (C_7, C_8, C_{11}, C_{12}); 62.1, 62.1 (C_{10}, C_{14}); 18.9 (C_{18}); 11.5 (C_{17}). MS (ESI) calcd for $C_{25}H_{41}IO_6Si$ $(M + I)^+$ 592.6; found, 593.0

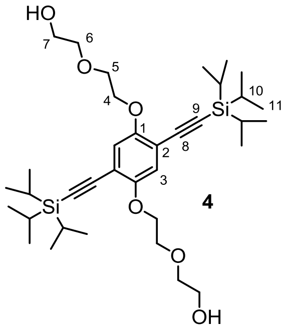

4

^1H NMR (400 MHz, $CDCl_3$) δ 6.89 (2H, s, H_3), 4.15–4.09 (4H, m, H_4), 3.85–3.80 (4H, m, H_5), 3.74–3.67 (4H, m, H_7), 3.66–3.59 (4H, m, H_6), 1.29–0.91 (6H, m, H_{10}), 1.10–1.12 (38H, m, H_{11}); ^{13}C NMR (100 MHz, $CDCl_3$) δ 154.1 (C_1); 118.2 (C_3); 114.7 (C_2); 102.9 (C_8); 97.1 (C_9); 72.7(C_6); 69.9 (C_5); 69.3 (C_4); 62.1 (C_7); 18.9 (C_{11}); 11.6 (C_{10}); MS (ESI) calcd for $C_{36}H_{62}O_6Si_2Na$ $(M + Na)^+$ 670.0; found, 669.8

1,4-bis[2-(2-hydroxyethoxy)ethoxy]-2,5-diethynylbenzene (11)

To a solution of **4** (200 mg, 0.31 mmol, 1 equiv), in THF (3 mL) TBAF (1 M, 0.93 mL, 0.93 mmol, 3 equiv), was added. The resulting solution was stirred for 20 min at room temperature. The solvent was removed under reduced pressure and the crude was purified by flash chromatography (silica, chloroform with gradient of methanol from 0 to 15%) to afford 62 mg (62%) of pure product.

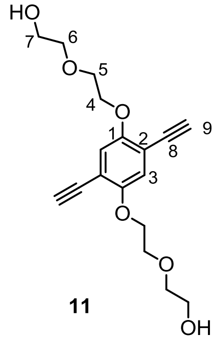

11

^1H NMR (400 MHz, $CDCl_3$) δ 6.98 (2H, s, H_3), 4.16–4.10 (4H, m, H_4), 3.90–3.82 (4H, m, H_5), 3.76–3.70 (4H, m, H_7), 3.68–3.62 (4H, m, H_6), 3.34 (2H,s, H_9); ^{13}C NMR (100 MHz, $CDCl_3$) δ 154.2 (C_1); 118.4 (C_3); 113.8 (C_2); 83.2 (C_8); 79.6 (C_9); 72.7 (C_6); 69.6 (C_5); 69.5 (C_4); 62.0 (C_7); MS (ESI) calcd for $C_{18}H_{22}O_6Na$ $(M + Na)^+$ 357.4; found, 357.3

Rod 13

11 (50 mg, 0.151 mmol, 3 equiv), **3** (30 mg, 0.05 mmol, 1 equiv), $Pd(PPh_3)_4$, (5.8 mg, 0.005 mmol, 0.1 equiv), CuI (1 mg, 0.005 mmol, 0.1 equiv), and PPh_3 (2.6 mg, 0.01 mmol, 0.2 equiv) were placed into the reaction flask and dried under vacuum, then the reagents were dissolved by addition of toluene (0.6 mL) and Et_3N (0.2 mL) under nitrogen. The reaction was heated to 50 °C 14 h. The solvent was removed under reduced pressure and the resulting crude was purified by flash chromatography (chloroform with gradient of methanol from 0 to 15%) to afford 15.5 mg (50%) of product **13**.

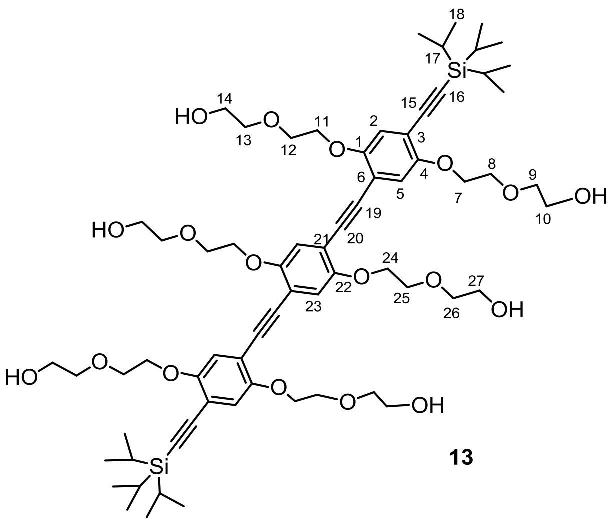

13

^1H NMR (400 MHz, CD_3OD) δ 7.16 (2H, s, H_2), 7.12 (2H, s, H_5), 7.05 (2H,s, H_{23}), 4.31–4.12 (12H, m, H_7, H_{11}, H_{24}), 3.97–3.80 (12H, m, H_8, H_{12}, H_{25}), 3.76–3.56 (24H, m, H_9, H_{10}, H_{13}, H_{14}, H_{26}, H_{27}), 1.35–0.98 (6H, m, H_{17}), 1.16–1.18 (36H, m, H_{18}); ^{13}C NMR (100 MHz, CD_3OD) δ 155.9, 155.2, 154.9 (C_4, C_1, C_{22}); 119.6, 119.1, 118.3 (C_3, C_6, C_{21}); 116.1, 116.0, 115.7 (C_5, C_2, C_{23}); 104.3 (C_{15}); 97.7 (C_{16}); 92.6, 92.6 (C_{19}, C_{20}); 74.3, 74.2, 74.1 (C_9, C_{13}, C_{26}); 71.2, 71.1, 71.0, 71.0, 70.5 (C_7, C_8, C_{11}, C_{12}, C_{24}, C_{25}); 65.5, 62.4 (C_{10}, C_{14}, C_{27}); 19.3 (C_{18}); 12.7 (C_{17}); MS (ESI) calcd for $C_{68}H_{102}NaO_{18}Si_2$ $(M + Na)^+$ 1286.7; found. 1286.0.

1,4-bis[2-(2-methoxyethoxy)ethoxy]-2-[trimethylsilyl]ethynyl-5-iodobenzene (5) and 1,4-bis[2-(2-methoxyethoxy)ethoxy]-2,5-bis[trimethylsilyl]ethynylbenzene (6)

The bis-iodinated compound **2** (1g, 1.77 mmol), was dissolved in THF (10 mL). ethynyltrimethylsilane (TMS-acetylene, 2.6 g, 2.65 mmol), $PdCl_2(PPh_3)_2$ (12.4 mg, 0.17 mmol, 0.1 equiv), CuI (6.74 mg, 0.35, 0.2 equiv), Et_3N (1 mL, 4 equiv) were added to the solution. The mixture was heated under microwave irradiation at 60 °C for 20 min.

After removal of the solvent the final products were purified by column chromatography to afford compound **5** (0.48 mg, 35%) and compound **6** (0.33 mg, 53%). The NMR data were in accord with those reported in literature [5].

General Sonogashira reaction for the preparation of compounds 7, 9, 14, 16, 18.

The free alkyne compound (1 equiv) was dissolved in THF and the monoiodinated compound **5** (1.2 equiv), $PdCl_2(PPh_3)_2$ (0.1 equiv), CuI (0.1 equiv), Et_3N (4 equiv) were added. The mixture was heated under microwave irradiation at 60 °C for 20 min. After evaporation of the solvent, the desired product was purified by column chromatography.

General TMS-cleavage reaction for the preparation of compounds 8, 12, 15, 17, 19.

A mixture of a double TMS-protected compound (1 equiv) and K_2CO_3 (2 equiv) in the mixed solvents MeOH/DCM (3/1) was stirred for 45 min at rt. The reaction mixture was washed with H_2O. The aqueous layer was extracted with DCM. The combined organic layers were dried over Na_2SO_4, filtered and concentrated in vacuo to afford the desired product.

1,4-bis[2-(2-methoxyethoxy)ethoxy]-2-[*tert*-butyldimethylsilyl]ethynyl-5-[trimethylsilyl]ethynylbenzene (7)

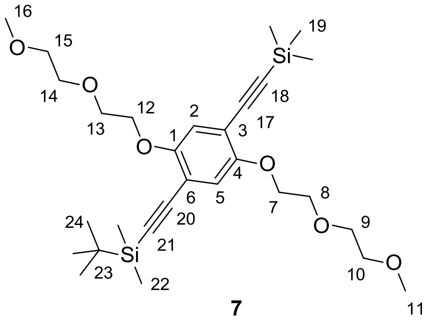

7

0.49 mg, 81%: ^{1}H NMR (300 MHz, $CDCl_3$) δ 6.90 (2H, s, H_2, H_5), 4.15–4.06 (4H, m, H_7, H_{12}), 3.89–3.80 (4H, m, H_8, H_{13}), 3.79–3.69 (4H, m, H_9, H_{14}), 3.58–3.50 (4H, m, H_{10}, H_{15}), 3.38 (6H, s, H_{11}, H_{16}), 0.99 (9H, s, H_{24}), 0.24 (9H, s, H_{19}) 0.16 (6H, s, H_{22}); ^{13}C NMR (75.5 MHz, $CDCl_3$) δ 153.9, 153.8 (C_1, C_4), 117.9, 117.5 (C_3, C_6), 114.2, 114.1 (C_2, C_5), 101.4(C_{20}), 100.9 (C_{17}), 100.1 (C_{21}), 98.7 (C_{18}), 72.1, 72.0 (C_{10}, C_{15}), 71.1, 70.9 (C_9, C_{14}), 69.7, 69.6 (C_8, C_{13}), 69.2 (C_7, C_{12}), 59.0 (C_{11}, C_{16}), 26.2 (C_{23}), 16.7 (C_{24}), −0.1 (C_{19}), −4.7 (C_{22}); MS (ESI) *m*/*z* calcd. for $C_{29}H_{49}O_6Si_2$ $(M + H)^+$ 549.30; found, 549.75.

1,4-bis[2-(2-methoxyethoxy)ethoxy]-2-[*tert*-butyldimethylsilyl]ethynyl-5-ethynylbenzene (8)

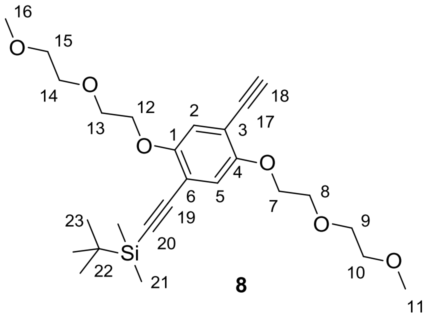

8

786 mg, 78%: ^{1}H NMR (300 MHz, $CDCl_3$) δ 6.93 (1H, s, H_2), 6.91 (1H, s, H_5), 4.04–4.17 (4H, m, H_7, H_{12}), 3.78–3.88 (4H, m, H_8, H_{13}), 3.63–3.74 (4H, m, H_9, H_{14}), 3.47–3.57 (4H, m, H_{10}, H_{15}), 3.35 (6H, s, H_{11}, H_{16}), 3.29 (1H, s, H_{18}), 0.97 (9H, s, H_{23}), 0.15 (6H, s, H_{21}); ^{13}C NMR (75.5 MHz, $CDCl_3$) δ 153.9 (C_1, C_4), 118.0, 117.8 (C_3, C_6), 114.6, 113.1 (C_2, C_5), 101.3 (C_{19}), 98.8 (C_{20}), 82.6 (C_{17}), 79.7 (C_{18}), 72.0 (C_{10}, C_{15}), 70.9, 69.7, 69.6, 69.5, 69.2 (C_7, C_8, C_9, C_{12}, C_{13}, C_{14}), 59.0 (C_{11}, C_{16}), 26.1 (C_{22}), 16.7 (C_{23}), -4.7 (C_{21}); MS (ESI) *m*/*z* calcd. for $C_{26}H_{41}O_6Si$ $(M + H)^+$ 477.26; found, m/z 477.25.

Rod 9

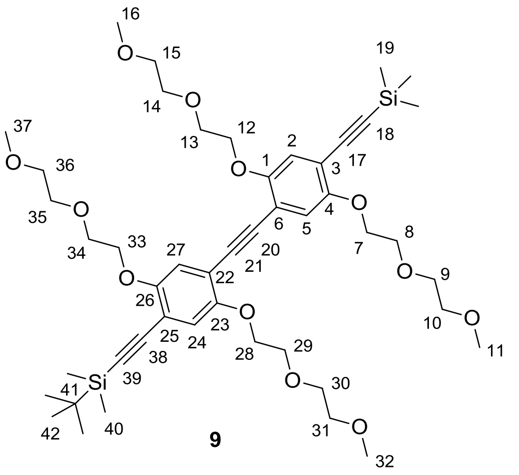

9

113 mg, 79%: ^{1}H NMR (300 MHz, $CDCl_3$) δ 6.99 (1H, s, H_2), 6.96 (1H, s, H_5), 6.95 (1H, s, H_{24}), 6.94 (1H, s, H_{27}), 4.08–4.22 (8H, m, H_7, H_{12}, H_{28}, H_{33}), 3.82–3.92 (8H, m, H_8, H_{13}, H_{29}, H_{34}), 3.70–3.81 (8H, m, H_9, H_{14}, H_{30}, H_{35}), 3.45–3.60 (8H, m, H_{10}, H_{15}, H_{31}, H_{36}), 3.33–3.40 (12H, H_{11}, H_{16}, H_{32}, H_{37}), 0.99 (9H, s, H_{41}), 0.26 (9H, s, H_{19}), 0.18 (6H, s, H_{40}); ^{13}C NMR (75.5 MHz, $CDCl_3$) δ 154.1, 154.0, 153.4, 153.3 (C_1, C_4, C_{23}, C_{26}), 118.5, 118.3, 117.3, 117.0 (C_3, C_6, C_{25}, C_{28}), 114.6, 114.5, 114.2, 114.1 (C_2, C_5, C_{24}, C_{27}), 101.5 (C_{38}), 100.9 (C_{39}), 100.4 (C_{17}), 98.8 (C_{18}), 91.3, 91.1 (C_{20}, C_{21}), 72.0 (C_{10}, C_{15}, C_{31}, C_{36}), 71.1, 70.9, 69.7, 69.6, 69.2 (C_7, C_8, C_9, C_{12}, C_{13}, C_{14}, C_{28}, C_{29}, C_{30}, C_{33}, C_{34}, C_{35}), 59.0 (C_{11}, C_{16}, C_{32}, C_{37}), 26.2 (C_{41}), 16.7 (C_{42}), −0.1 (C_{19}), −4.6 (C_{40}); MS (ESI) *m*/*z* calcd. for $C_{47}H_{72}O_{12}Si_2Na$ $(M + Na)^+$ 907.44; found, 907.50 .

Rod 10

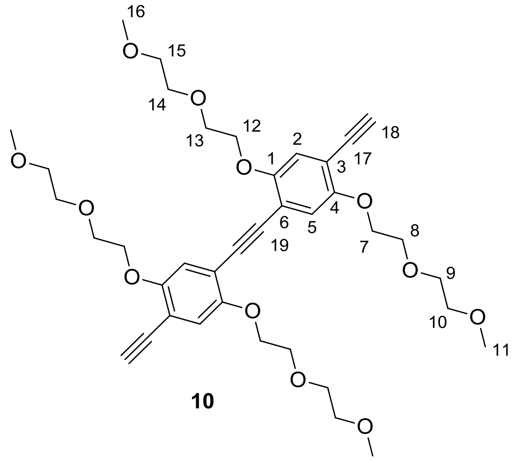

10

The protected di-unit spacer **9** (140 mg, 0.16 mmol) was dissolved in THF (10 mL) and a solution of *tetra*-*n*-butylammonium fluoride (TBAF) (100 mg, 0.32 mmol) in THF (5 mL) was added dropwise. After 2 h at rt, water (15 mL) was added to quench the reaction. The compound was extracted with DCM. The organic layer were dried over Na_2SO_4 and filtered. The compound was purified by column chromatography to give product **10** as a yellow solid (94 mg, 84%).

^{1}H NMR (300 MHz, $CDCl_3$) δ 7.03 (2H, s, H_2), 7.01 (2H, s, H_5), 4.23–4.14 (8H, m, H_7, H_{12}), 3.91–3.85 (8H, m, H_8, H_{13}), 3.79–3.71 (8H, m, H_9, H_{13}), 3.58–3.48 (8H, m, H_{10}, H_{14}), 3.39–3.33 (14H H_{11}, H_{16}, H_{18}); ^{13}C NMR (75.5 MHz, $CDCl_3$): δ = 154.2, 153.4 (C_1, C_4), 118.8, 117.4 (C_3, C_6), 115.0, 113.1 (C_2, C_5), 91.1 (C_{17}, C_{19}), 82.7 (C_{18}), 72.0 (C_{10}, C_{15}), 71.0, 69.7, 69.6, 69.5 (C_7, C_8, C_9, C_{12}, C_{13}, C_{14}), 59.0 (C_{11}, C_{16}); MS (ESI) *m*/*z* calcd. for $C_{38}H_{51}O_{12}$ $(M + H)^+$ 699.33; found, 698.75

Rod 16

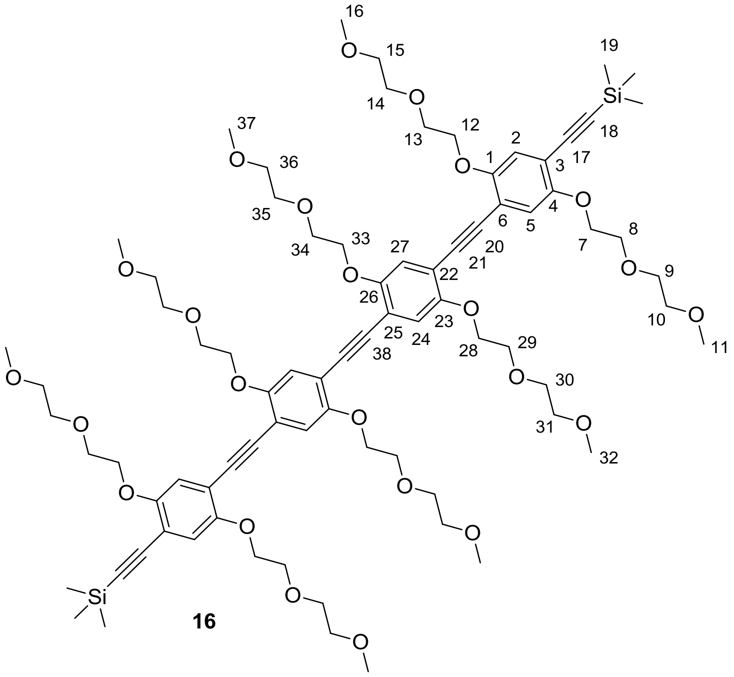

16

113 mg, 73%: ^{1}H NMR (300 MHz, $CDCl_3$) δ 7.01 (4H, d, H_{24}, H_{27}), 6.98 (2H, s, H_2), 6.94 (2H, s, H_5), 4.20–4.05 (16H, m, H_7, H_{12}, H_{28}, H_{33}), 3.92 -3.82 (8H, m, H_8, H_{13}, H_{29}, H_{34}), 3.80–3.70 (16H, m, H_9, H_{14}, H_{30}, H_{35}), 3.60–3.45 (16H, m, H_{10}, H_{15}, H_{31}, H_{36}), 3.35–3.29 (24H, H_{11}, H_{16}, H_{32}, H_{37}), 0.23 (18H, s, H_{19}); ^{13}C NMR (75.5 MHz, $CDCl_3$) δ 154.1, 153.5, 153.3 (C_1, C_4, C_{23}, C_{26}), 118.9, 117.9, 117.8, 117.3 (C_3, C_6, C_{25}, C_{28}), 114.6, 114.5, 114.4 (C_2, C_5, C_{24}, C_{27}), 100.9, 100.4 (C_{17}, C_{18}), 91.5, 91.4, 91.3 (C_{20}, C_{21}, C_{38}), 72.0 (C_{10}, C_{15}, C_{31}, C_{36}), 71.0, 69.6 (C_7, C_8, C_9, C_{12}, C_{13}, C_{14}, C_{28}, C_{29}, C_{30}, C_{33}, C_{34}, C_{35}), 59.0 (C_{11}, C_{16}, C_{32}, C_{37}),−0.1 (C_{19}); MS (ESI) *m/z* calcd. for $C_{80}H_{114}O_{24}Si_2Na$ $(M + Na)^+$ 1537.71; found, 1538.25.

Rod 17

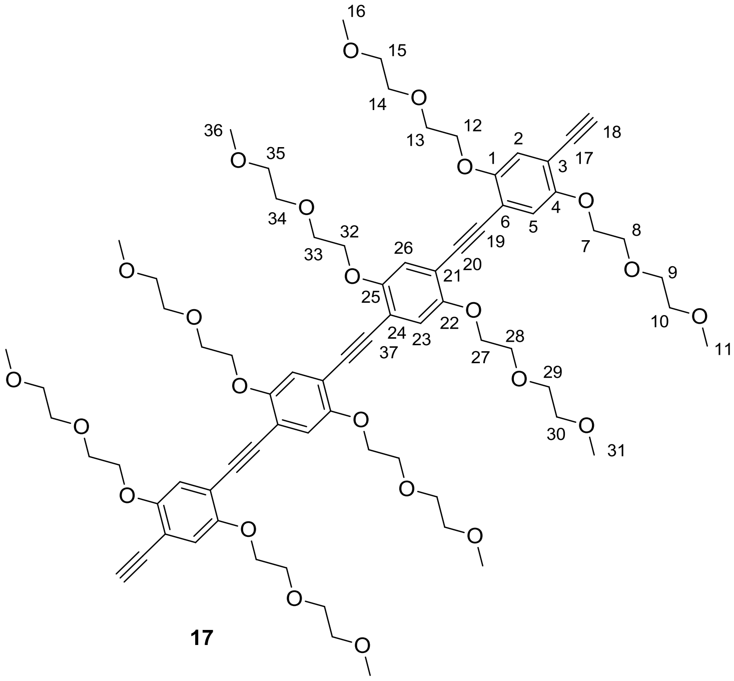

17

40.6 mg, 76%: ^{1}H NMR (300 MHz, $CDCl_3$) δ 7.03 (6H, s, H_5, H_{23}, H_{26}), 7.01 (2H, s, H_2), 4.23–4.17 (16H, m, H_7, H_{12}, H_{27}, H_{32}), 3.95–3.82 (16H, m, H_8, H_{13}, H_{28}, H_{33}), 3.79–3.70 (16H, m, H_9, H_{14}, H_{29}, H_{34}), 3.58–3.45 (16H, m, H_{10}, H_{15}, H_{30}, H_{35}), 3.39–3.35 (26H, s, H_{11}, H_{16}, H_{18}, H_{31}, H_{36}); ^{13}C NMR (75.5 MHz, $CDCl_3$) δ 154.2, 153.5, 153.4 (C_1, C_4, C_{22}, C_{25}), 118.9, 117.9, 117.8, 117.5 (C_3, C_6, C_{24}, C_{27}), 115.0, 114.6, 114.5 (C_2, C_5, C_{23}, C_{26}), 91.5, 91.4, 91.2 (C_{19}, C_{20}, C_{37}), 82.7 (C_{17}), 79.7 (C_{18}), 72.0 (C_{10}, C_{15}, C_{30}, C_{35}), 71.0, 69.6 (C_7, C_8, C_9, C_{12}, C_{13}, C_{14}, C_{27}, C_{28}, C_{29}, C_{32}, C_{33}, C_{34}), 59.0 (C_{11}, C_{16}, C_{31}, C_{36}); MS (ESI) *m/z* calcd. for $C_{74}H_{99}O_{24}$ (M + H)$^+$ 1371.65; found, 1372.14.

Rod 18

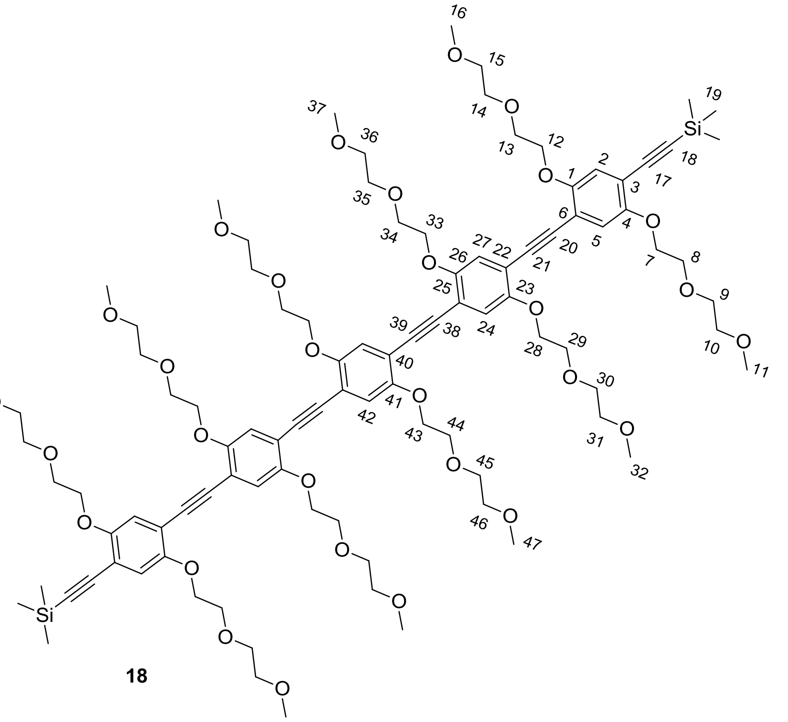

18

586 mg, 70%: ^{1}H NMR (300 MHz, $CDCl_3$) δ 7.05 (6H, s, H_{24}, H_{27}, H_{42}), 7.01 (2H, s, H_2), 6.98 (2H, s, H_5), 4.10–4.29 (20H, m, H_7, H_{12}, H_{28}, H_{33}, H_{43}), 3.83–3.97 (20H, m, H_8, H_{13}, H_{29}, H_{34}, H_{44}), 3.67–3.82 (20H, m, H_9, H_{14}, H_{30}, H_{36}, H_{45}), 3.45–3.61 (20H, m, H_{10}, H_{15}, H_{31}, H_{36}, H_{46}), 3.39–3.34 (30H, s, H_{11}, H_{16}, H_{32}, H_{37}, H_{47}), 0.26 (16H, s, H_{19}); ^{13}C NMR (75.5 MHz, $CDCl_3$) δ 154.1, 153.5, 153.3 (C_1, C_4, C_{23}, C_{26}, C_{41}), 118.4, 117.9, 117.3 (C_3, C_6, C_{25}, C_{28}, C_{40}), 114.5, 114.2 (C_2, C_5, C_{24}, C_{27}, C_{42}), 100.9, 100.5 (C_{17}, C_{18}), 91.4, 91.3 (C_{20}, C_{21}, C_{38}, C_{39}), 72.0 (C_{10}, C_{15}, C_{31}, C_{36}, C_{46}), 71.1, 69.6 (C_7, C_8, C_9, C_{12}, C_{13}, C_{14}, C_{28}, C_{29}, C_{30}, C_{33}, C_{34}, C_{35}, C_{43}, C_{44}, C_{45}) 59.0 (C_{11}, C_{16}, C_{32}, C_{37}, C_{47}), −0.1 (C_{19}); MS (ESI) *m/z* calcd. for $C_{98}H_{138}O_{30}Si_2Na$ (M + Na)$^+$ 1873.87; found, 1874.98.

Rod 19

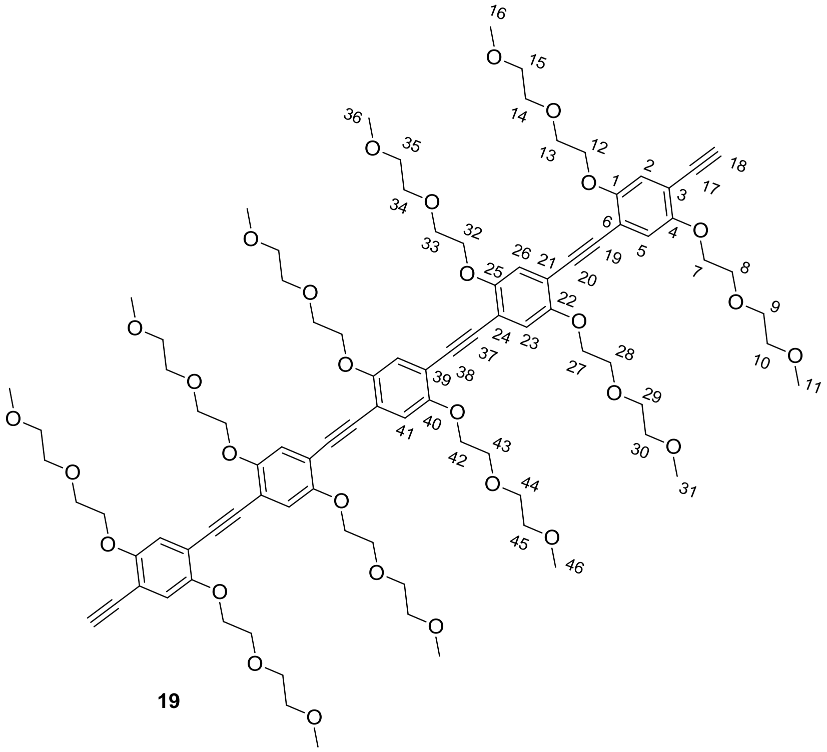

19

260 mg, 75%: ^{1}H NMR (300 MHz, $CDCl_3$) δ 7.04 (8H, s, H_2, H_{24}, H_{27}, H_{42}), 7.01 (2H, s, H_5), 4.13–4.25 (20H, m, H_7, H_{12}, H_{27}, H_{32}, H_{42}), 3.84–3.93 (20H, m, H_{13}, H_{28}, H_{33}, H_{43}), 3.71–3.79 (20H, m, H_9, H_{14}, H_{29}, H_{34}, H_{44}), 3.46–3.58 (20H, m, H_{10}, H_{15}, H_{30}, H_{35}, H_{45}), 3.38–3.33 (32H, m, H_{11}, H_{16}, H_{18}, H_{31}, H_{36}, H_{46}); ^{13}C NMR (75.5 MHz, $CDCl_3$) δ 154.2, 153.6, 153.4 (C_1, C_4, C_{22}, C_{25}, C_{40}), 118.8, 117.9, 117.4 (C_3, C_6, C_{21}, C_{24}, C_{39}), 114.6, 114.4, 113.1 (C_2, C_5, C_{23}, C_{26}, C_{41}), 91.5, 91.3, 91.2 (C_{17}, C_{18}, C_{19}, C_{20}, C_{37}), 82.6 (C_{17}), 79.8 (C_{18}), 72.0 (C_{10}, C_{15}, C_{30}, C_{35}, C_{45}), 71.0, 69.7, 69.5 (C_7, C_8, C_9, C_{12}, C_{13}, C_{14}, C_{27}, C_{28}, C_{29}, C_{30}, C_{32}, C_{33}, C_{34}, C_{42}, C_{43}, C_{44}), 59.0; MS (ESI) *m/z* calcd. for $C_{92}H_{122}O_{30}Na$ $(M + Na)^+$ 1729.79; found, 1730.10.

Compound 21

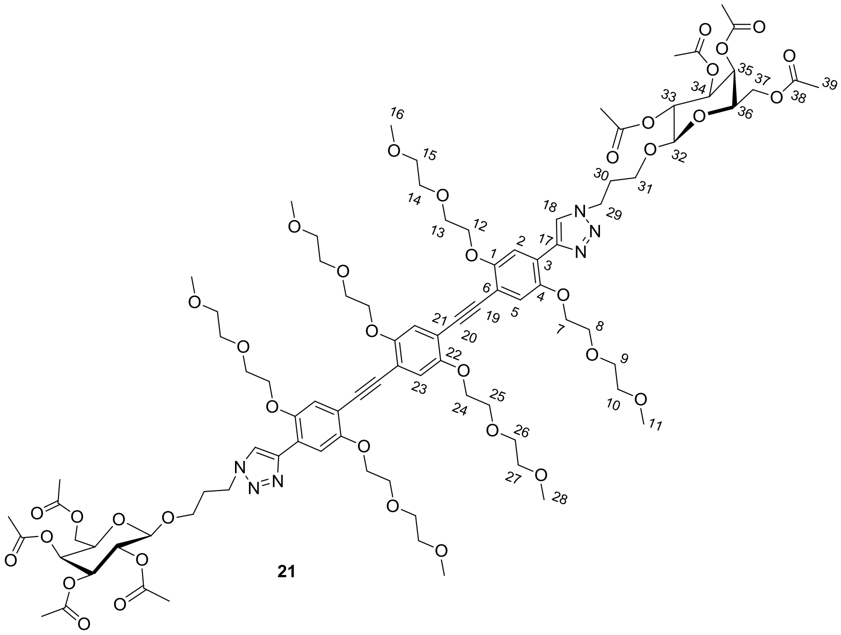

21

A mixture of **15** (68 mg, 66 µmol), galactose azide **20** (62 mg, 145 µmol), $CuSO_4 \cdot 5H_2O$ (7 mg, 26 µmol), sodium ascorbate (10 mg, 53 µmol) in DMF (1.8 mL) and H_2O (0.2 mL) was stirred in the microwave for 40 min at 80 °C. The solvent was removed in vacuo. The yellow residue (130 mg) was dissolved in DCM (15 mL) and washed three times with H_2O and brine. The organic layer was dried over Na_2SO_4, filtered and concentrated in vacuo. After column chromatography the pure product **21** was obtained as a crystalline yellow solid (102 mg, 85%).

^{1}H NMR (300 MHz, $CDCl_3$) δ 8.35 (2H, s, H_{18}), 7.96 (2H, s, H_2), 7.08 (2H, s, H_5), 7.07 (2H, s, H_{23}), 5.41 (2H, d, H_{35}, $J_{35\text{-}34}$ = 3.4 Hz), 5.27 (2H, dd, H_{33}, $J_{33\text{-}32}$ = 8.0 Hz, $J_{33\text{-}34}$ = 10.2 Hz), 5.03 (2H, dd, H_{34}, $J_{34\text{-}35}$ = 3.3 Hz, $J_{34\text{-}33}$ = 10.5 Hz), 4.62–4.38 (4H, m, H_{29}, H_{32}), 4.34 (4H, m, H_{24}), 4.29-4.20 (8H, m, H_7, H_{12}), 4.14 (4H, d, H_{37}, $J_{35\text{-}34}$ =6.94 Hz), 3.98–3.81 (18H, m, H_8, H_{13}, H_{25}, H_{31}, H_{36}), 3.83–3.70 (12H, m, H_9, H_{14}, H_{26}), 3.62–3.50 (12H, m, H_{10}, H_{15}, H_{27}), 3.38–3.35 (26H, m, H_{11}, H_{16}, H_{28}), 2.37–2.19 (4H, m, H_{30}), 2.16, 2.12, 2.03, 2.00 (24H, s, H_{39}). ^{13}C NMR (75.5 MHz, $CDCl_3$): 170.6, 170.2, 170.1, 169.7 (C_{38}), 154.2, 153.5 (C_1, C_{22}), 148.6 (C_{17}), 142.7 (C_4), 124.3 (C_{18}), 121.6 (C_3), 117.9, 117.7 (C_5, C_{23}), 114.6 (C_2), 112.7, 111.63 (C_6, C_{21}), 101.4 (C_{32}), 91.7, 90.4 (C_{19}, C_{20}), 72.0, 71.0, 70.8, 70.7, 70.3, 69.7, 69.5, 68.8, 67.8 (C_7, C_8, C_9, C_{10}, C_{12}, C_{13}, C_{14}, C_{15}, C_{25}, C_{25}, C_{26}, C_{27}, C_{33}, C_{34}, C_{36}), 67.0 (C_{35}), 66.12 (C_{31}), 61.2 (C_{37}), 58.9 (C_{11}, C_{16}, C_{28}), 46.5 (C_{29}), 30.4 (C_{30}), 20.9-20.5 (C_{39}); MS (ESI) *m/z* calcd. for $C_{90}H_{124}N_6O_{38}$ (M + 2H)$^{2+}$ 949.40; found, 949.70.

Compound 22

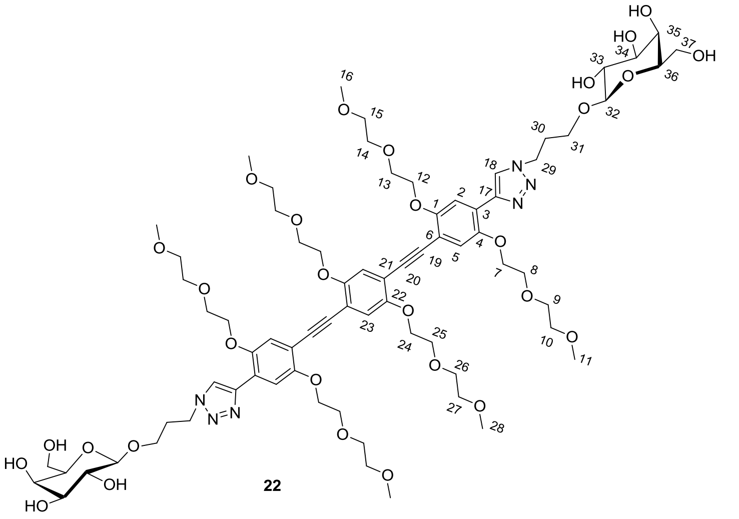

22

Compound **21** (67 mg, 38 µmol) was dissolved in a flask with MeOH (3 mL), and NaOMe (50 µL, 0.54 mmol) was added. The reaction mixture was stirred for 1 h, neutralized with Dowex H^+ and filtered. The solution was concentrated, and preparative HPLC was performed for purification to afford **22** (23 mg, 41%) as a yellow solid.

^{1}H NMR (300 MHz, CD_3OD) δ 8.49 (2H, s, H_{18}), 7.81 (2H, s, H_2), 7.18 (2H, s, H_5), 7.13 (2H, s, H_{23}), 4.64 (4H, t, H_{29}, $J_{29,30}$ = 6.82 Hz), 4.31 (4H, m, H_{24}), 4.25–4.18 (10H, m, H_7, H_{12}, H_{32}), 3.98–3.85, (16H, m, H_8, H_{13}, H_{25}, H_{31a}, H_{35}), 3.83–3.69 (16H, m, H_9, H_{14}, H_{26}, H_{37}), 3.64–3.46 (20H, m, H_{10}, H_{15}, H_{27}, H_{31b}, H_{33}, H_{34}, H_{36}), 3.32–3.28 (18H, m, H_{11}, H_{16}, H_{28}), 2.26 (4H, quint, H_{30}, $J_{30,29}$ = 6.82 Hz); ^{13}C NMR (75.5 MHz, CD_3OD) δ 153.9 (C_{22}), 153.6 (C_4), 149.1 (C_1), 142.2 (C_{17}), 125.1 (C_{18}), 120.9 (C_{21}), 117.6 (C_6), 117.0 (C_3), 114.5 (C_{23}), 113.1 (C_5), 111.3 (C_2), 103.7 (C_{32}), 91.3, 90.3 (C_{19}, C_{20}), 75.3 (C_{34}), 73.6 (C_{33}), 71.7, 71.1, 70.5, 69.8, 69.5, 69.4, 69.3, 68.9, 67.9 (C_7, C_8, C_9, C_{10}, C_{12}, C_{13}, C_{14}, C_{15}, C_{24}, C_{25}, C_{26}, C_{27}, C_{35}, C_{36}), 65.7 (C_{31}), 61.1 (C_{37}), 57.8 (C_{11}, C_{16}, C_{28}), 46.7 (C_{29}), 30.3 (C_{30}); HRMS (MALDI TOF/TOF) *m/z* calcd. for $C_{74}H_{109}N_6O_{30}$ $(M + H)^+$ 1561,7188; found, 1561.7128

Compound 24

To a solution of **13** (5.5 mg, 0.0043 mmol, 1 equiv) in THF (0.6 mL) a 1 M solution of TBAF (1 drop, cca 10 µL) was added. The reaction was stirred at room temperature. After 1 h TLC (DCM/MeOH 8:2) indicated no starting material. Then, to the reaction mixture the following reagents were added in the following order: water (0.6 mL), TBTA (0.46 mg, 0.0008 mmol, 0.2 equiv), copper(II) sulfate pentahydrate (0.1 mg, 0.0004 mmol, 0.1 equiv), sodium ascorbate (0.34 mg, 0.0017 mmol, 0.4 equiv) and finally **23** (5 mg, 0.0108 mmol, 2.5 equiv). The reaction mixture was stirred at room temperature under a nitrogen atmosphere in the dark. After 2 h TLC (silica, hex/EA 8:2 and C18, H_2O/MeOH 1:1) indicated no **13**, one major and one minor product. Another portion of sodium ascorbate (0.4 equiv) was added and the mixture was stirred for another 16 h. The reaction was charged to a column in order to purify by size-exclusion chromatography (Sephadex LH20, MeOH). The isolated product was further purified by reverse phase chromatography (C18, water with gradient of methanol from 0% to 100%) to afford 6.2 mg (76%) of pure product.

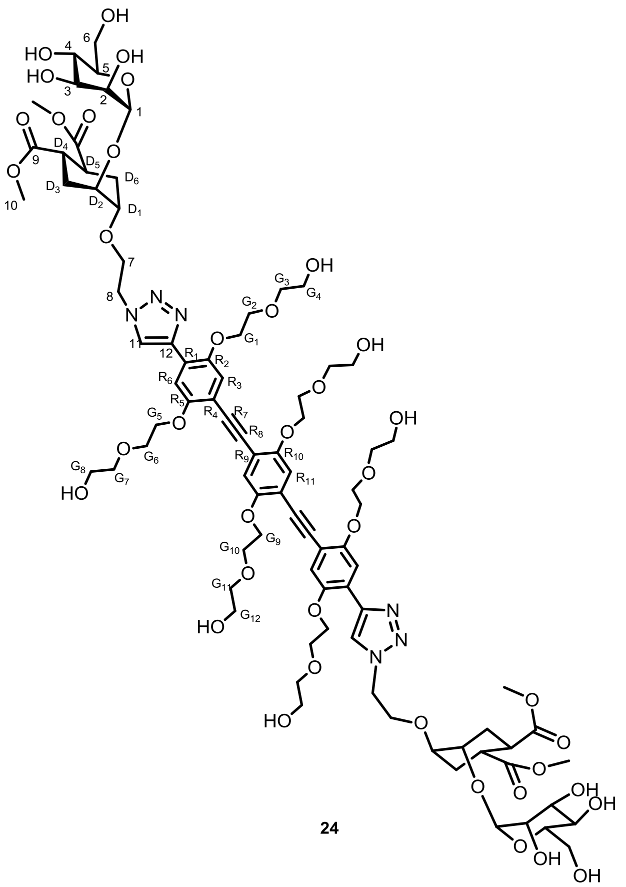

24

^{1}H NMR (400 MHz, CD_3OD) δ 8.63 (s, 2H, H_{11}), 7.88 (s, 2H, R_3), 7.24 (s, 2H, R_6), 7.18 (s, 2H, R_{11}), 4.89 (br s, 2H, H_1), 4.68 (t, 4H, H_8, $J_{8\text{-}7}$ = 4.7 Hz), 4.39–4.31 (m, 4H, G_9), 4.31–4.22 (m, 8H, G_1, G_5), 4.02–3.90 (m, 16H, G_2, G_6, G_{10}, H_7), 3.89–3.80 (m, 4H, H_{6a}, D_2), 3.80–3.75 (m, 6H, H_2, G_{12}), 3.75–3.59 (m, 26H, H_{6b}, D_1, H_3, G_3, G_4, G_7, G_8, G_{11}), 3.59–3.40 (m, 16H, H_{10}, H_5, H_4), 2.81–2.56 (m, 4H, D_4, D_5), 2.04–1.88 (m, 4H, D_{3equiv}, D_{6equiv}), 1.77–1.60 (m, 2H, D_{3ax} or D_{6ax}), 1.55–1.39 (m, 2H, D_{3ax} or D_{6ax}); ^{13}C NMR (100 MHz, CD_3OD) δ 177.1, 176.6 (C_9); 155.5, 155.1 (R_{10}, R_5); 150.8 (R_2); 143.7 (C_{12}); 127.5 (C_{11}); 122.6 (R_1); 119.3 (R_{11}); 118.5 (R_6); 116.1, 114.6 (R_4, R_9); 113.1 (R_3); 100.6 (C_1); 92.8, 91.7 (R_7, R_8); 75.8 (C_5); 75.7 (D_1); 74.3, 74.3, 73.9 (G_3, G_7, G_{11}); 72.6, 72.6 (C_2, C_3); 72.1 (D_2); 71.2, 71.1, 71.0, 71.0, 70.8 (G_1 ,G_2, G_5, G_6, G_9, G_{10}); 69.6 (C_7); 68.7 (C_4); 63.2 (C_6); 62.5, 62.5 62.4 (G_4 ,G_8, G_{12}); 52.5 (C_{10}); 51.7 (C_8); 46.8 (C_{15}); 40.3, 40.2 (D_4, D_5); 29.2, 28.2 (D_3, D_6); MS (HRMS) calcd for $C_{86}H_{120}N_6O_{40}$ (M + H)$^+$ 1876.75403; found, 1876.76014.

Synthesis of the two-unit spacer 30 starting from 3 (hydroxyl-terminated PEG chain)

The two-unit spacer **30** could be synthesized by using 2-methyl-3-butyn-2-ol (MEBYNOL) **27** to obtain the orthogonally protected intermediate **28**. Removal of 2-propanol was achieved with NaOH under heterogeneous conditions. This reaction is rather critical and it must be stopped before reaching full conversion. Longer reaction times lead to full deprotection of **28** to **11**. Nonetheless, under optimized conditions, **29** was isolated in 35% yields and 36% of **5** was recovered by chromatography (55% yield based on recovered starting material). As for **13**, also **30** was desilylated with TBAF immediately before in situ CuAAC reactions.

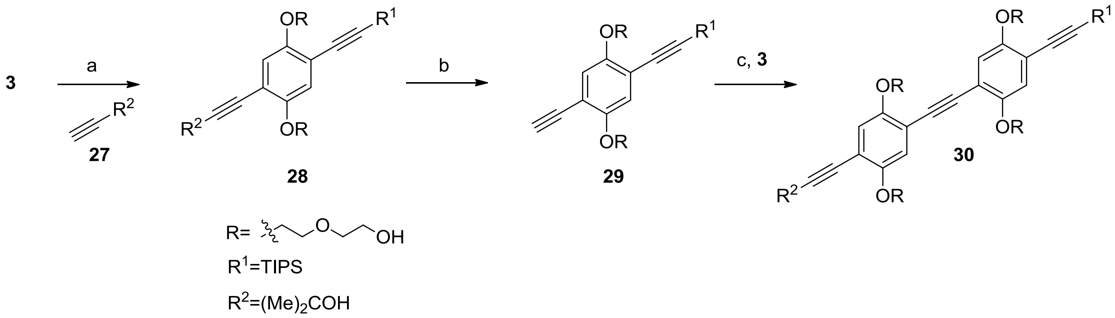

Scheme S1. Synthesis of two-units spacer. a) $PdCl_2(Ph_3P)_2$, CuI, DIPEA, THF, rt, 18 h, 85%; b) NaOH, toluene, 45 °C, 6 h, 35% (55%, based on recovered starting material).; c) $PdCl_2(Ph_3P)_2$, CuI, DIPEA, toluene, 3 h, 52%.

3 (50 mg, 0.084 mmol, 1 equiv), $PdCl_2(PPh_3)_2$ (3 mg, 0.004 mmol, 0.05 equiv), CuI (1 mg, 0.005 mmol, 0.06 equiv) and DIPEA (34 μL, 0.19 mmol, 2 equiv) were dissolved in dry THF (260 μL). **27** (10 μL, 0.11 mmol, 1.25 equiv) was added and the reaction mixture was stirred overnight at room temperature under nitrogen. The solvent was removed under reduced pressure and the resulting crude was purified by flash chromatography (hexane/AcOEt, with a gradient from 1:1 to 1:4) to afford 39 mg (85%) of product **28**.

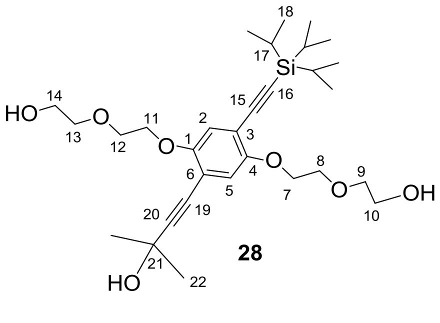

^{1}H NMR (400 MHz, $CDCl_3$) δ 6.90 (2H, 2s, H_2 and H_5), 4.15–3.95 (4H, m, H_7, H_{11}), 3.95–3.88 (2H, m, H_8) 3.86-3.82 (2H, m, H_{12}), 3.78–3.75 (2H, m, H_9), 3.70-3.74 (4H, m, H_{10}, H_{14}), 3.62–3.67 (2H, m, H_{13}) 2.55 (2H, bs, OH), 1.59 (6H, s, H_{22}), 1.18-1.10 (21H, m, H_{17}, H_{18}); ^{13}C NMR (100 MHz, $CDCl_3$) δ 154.3, 154.2 (C_4, C_1); 117.8, 117.5 (C_2, C_5); 114.4, 113.9 (C_3, C_6); 102.8 (C_{15}); 100.71 (C_{16}); 97.08 (C_{20}); 77.80 (C_{19}); 72.8, 72.3 (C_9, C_{13}); 69.87, 69.86 (C_8, C_{12}); 69.3 (C_7, C_{11}); 65.1 (C_{21}); 62.1, 61.6 (C_{10}, C_{14}); 31.5 (C_{22}); 18.9 (C_{18}); 11.6 (C_{17}); MS (ESI) calcd for $C_{30}H_{48}NaO_7Si$ $(M + Na)^+$ 571.32; found, 571.5.

28 (27 mg, 0.05 mmol, 1 equiv) was dissolved in dry toluene (500 μL) and solid NaOH (7 mg, 0.18 mmol, 3.6 equiv) was added. The reaction was stirred for 6 h at 45 °C under nitrogen and in the dark. The mixture was diluted with 97:3 $CHCl_3$:MeOH (2 mL) and **29** was isolated by flash chromatography (4:6 hexane:AcOEt, 8 mg, 35%. 10 mg of **28** was recovered).

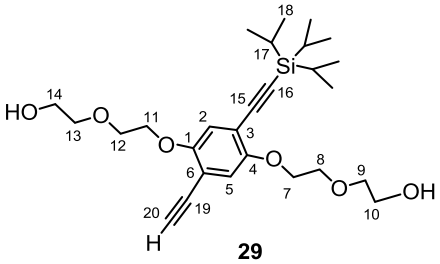

29

^{1}H NMR (400 MHz, $CDCl_3$) δ 6.96, 6.95 (2H, 2s, H_2 and H_5), 4.25–4.10 (4H, m, H_7, H_{11}), 3.90–3.80 (4H, m, H_8, H_{12}), 3.75–3.60 (8H, m, H_9, H_{10}, H_{13}, H_{14}), 3.35 (1H, s, H20), 2.05 (2H, bs, OH) 1.45–1.00 (21H, m, H_{17}, H_{18}); ^{13}C NMR (100 MHz, $CDCl_3$) δ 154.3, 154.2 (C_4, C_1); 118.5, 118.3 (C_2, C_5); 115.4, 113.3 (C_3, C_6); 102.6 (C_{15}); 97.7 (C_{16}); 83.1, 80.0 (C_{19}, C_{20}); 72.9 (C_9, C_{13}); 69.9, 69.8, 69.7, 69.4 (C_7, C_8, C_{11}, C_{12}); 62.2 (C_{10}, C_{14}); 19.4 (C_{18}); 11.6 (C_{17}); MS (ESI) calcd for $C_{27}H_{42}NaO_6Si$ $(M + Na)^+$ 513.7; found, 513.4.

29 (24 mg, 0.05 mmol, 1 equiv), **3** (30 mg, 0.05 mmol, 1 equiv), $PdCl_2(PPh_3)_2$ (2 mg, 0.003 mmol, 0.05 equiv) and CuI (1 mg, 0.005 mmol, 0.1 equiv) were dissolved in dry toluene (500 μL) and DIPEA (20 μL 0.11 mmol, 2 equiv) was added. The reaction mixture was stirred for 3 h at room temperature under nitrogen and in the dark. The solvent was removed under reduced pressure and the resulting crude was purified by flash chromatography (hexane/AcOEt, with a gradient from 1:4 to 1:9) to afford 25 mg (52%) of product **30**.

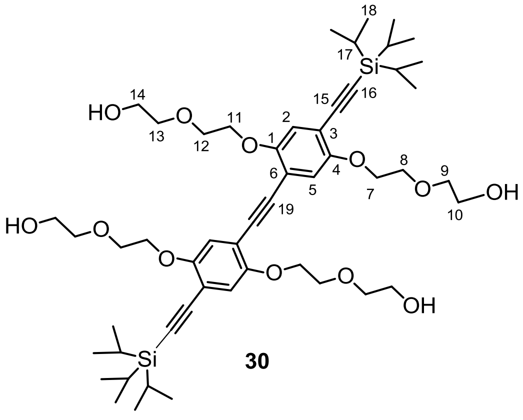

30

^{1}H NMR (400 MHz, CD_3OD) δ 7.11 (2H, s, H_2), 7.05 (2H, s, H_5), 4.31–4.12 (8H, m, H_7, H_{11}), 3.95–3.80 (8H, m, H_8, H_{12}), 3.74–3.60 (16H, m, H_9, H_{10}, H_{13}, H_{14}), 1.45-1.00 (42H, m, H_{17}, H_{18}); ^{13}C NMR (100 MHz, CD_3OD, HSQC) δ 119.1, 118.0 (C_5, C_2); 73.79, 73.76 (C_9, C_{13}); 70.59, 70.54 (C_7, C_8, C_{11}, C_{12}); 62.01, 61.96 (C_{10}, C_{14}); 18.83 (C_{18}); 12.20 (C_{17}); MS (ESI) calcd for $C_{52}H_{82}NaO_{12}Si_2$ $(M + Na)^+$ 977.53; found, 978.0.

Inhibition assay of LecA with compound 22

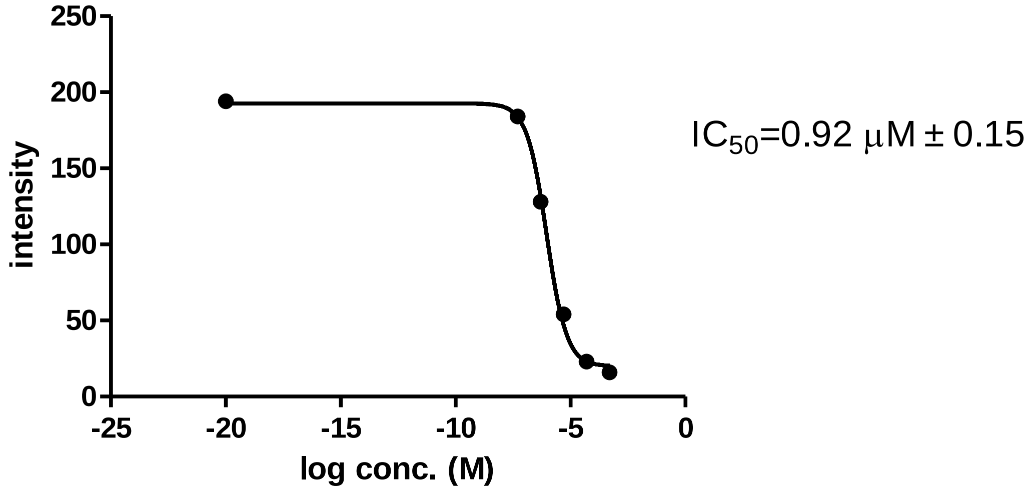

Compound **3** ^{1}H and ^{13}C NMR

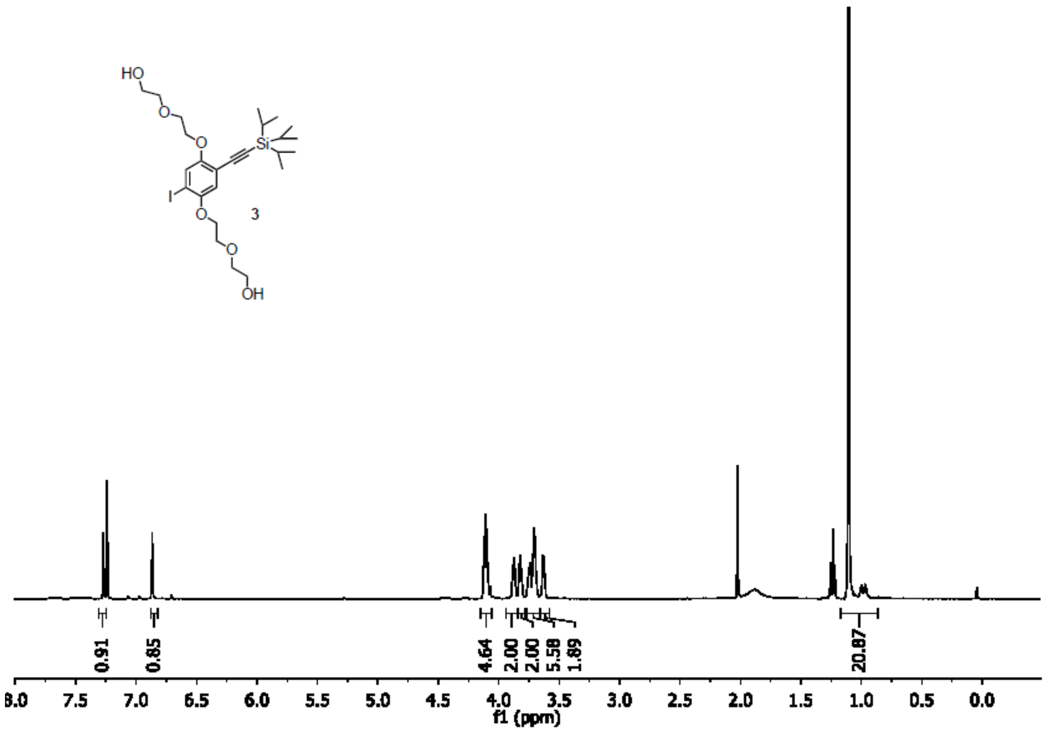

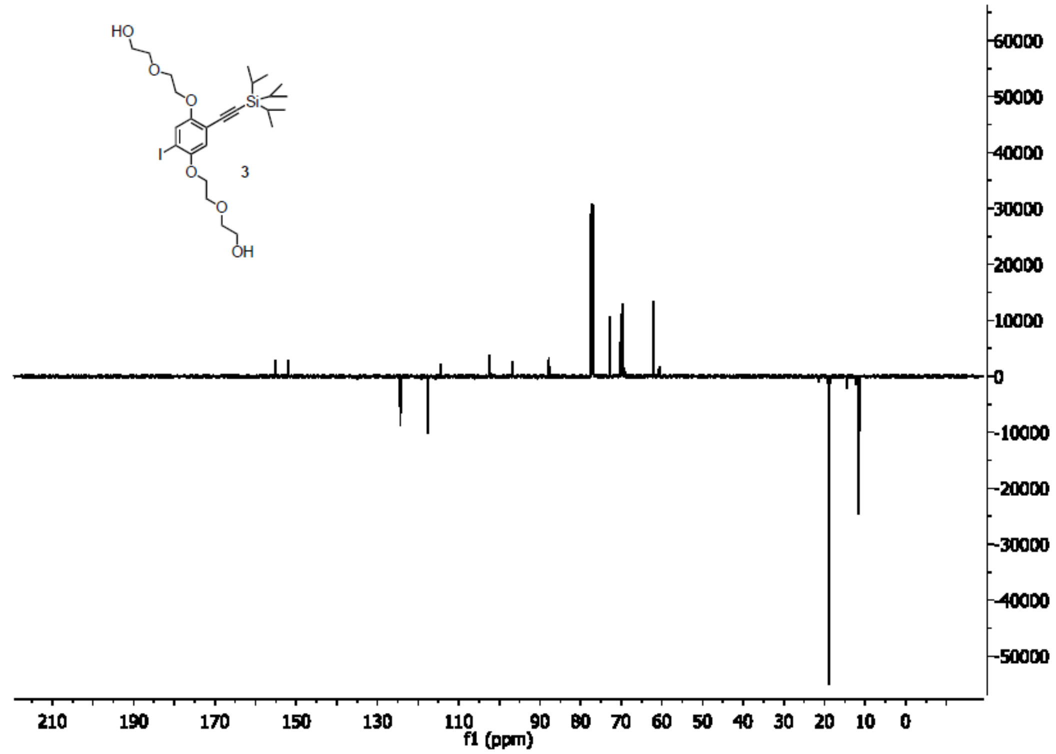

Compound **4** ^{1}H and ^{13}C NMR

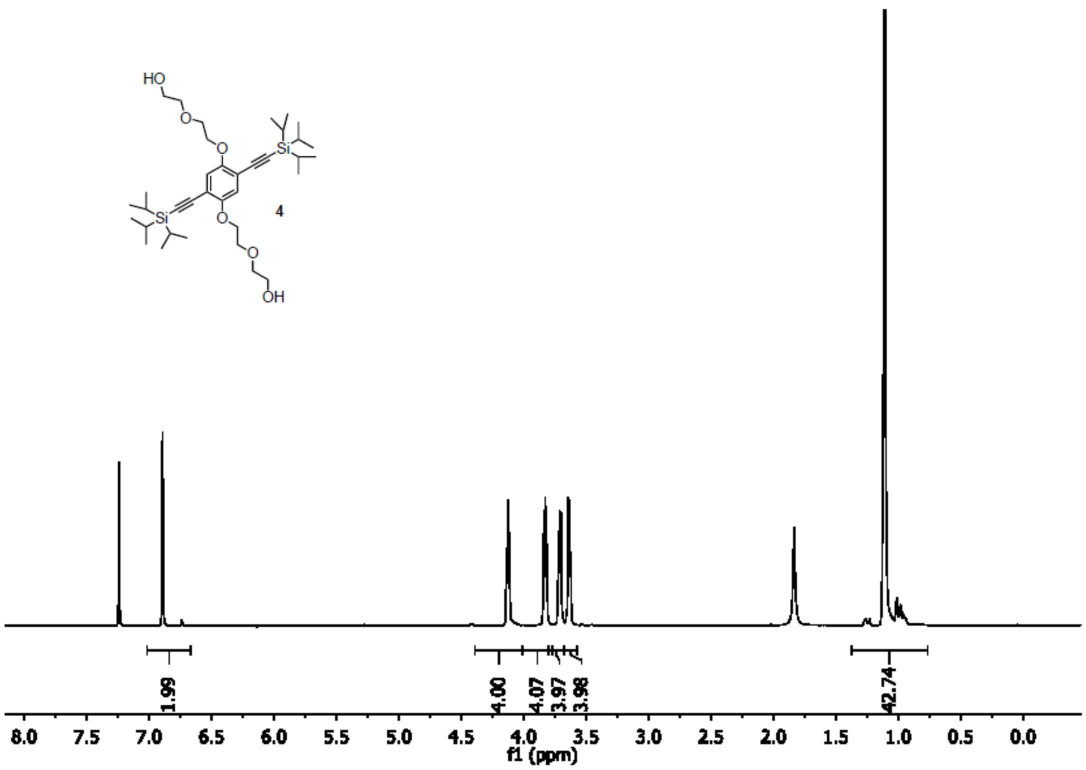

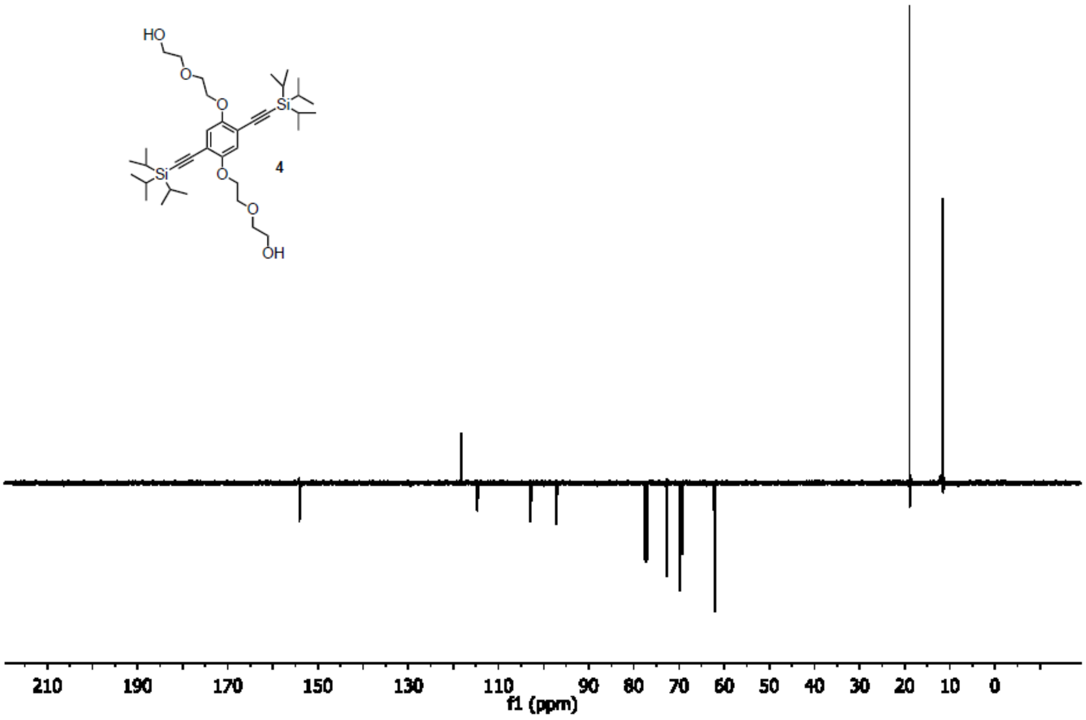

Compound **7** ^{1}H and ^{13}C NMR

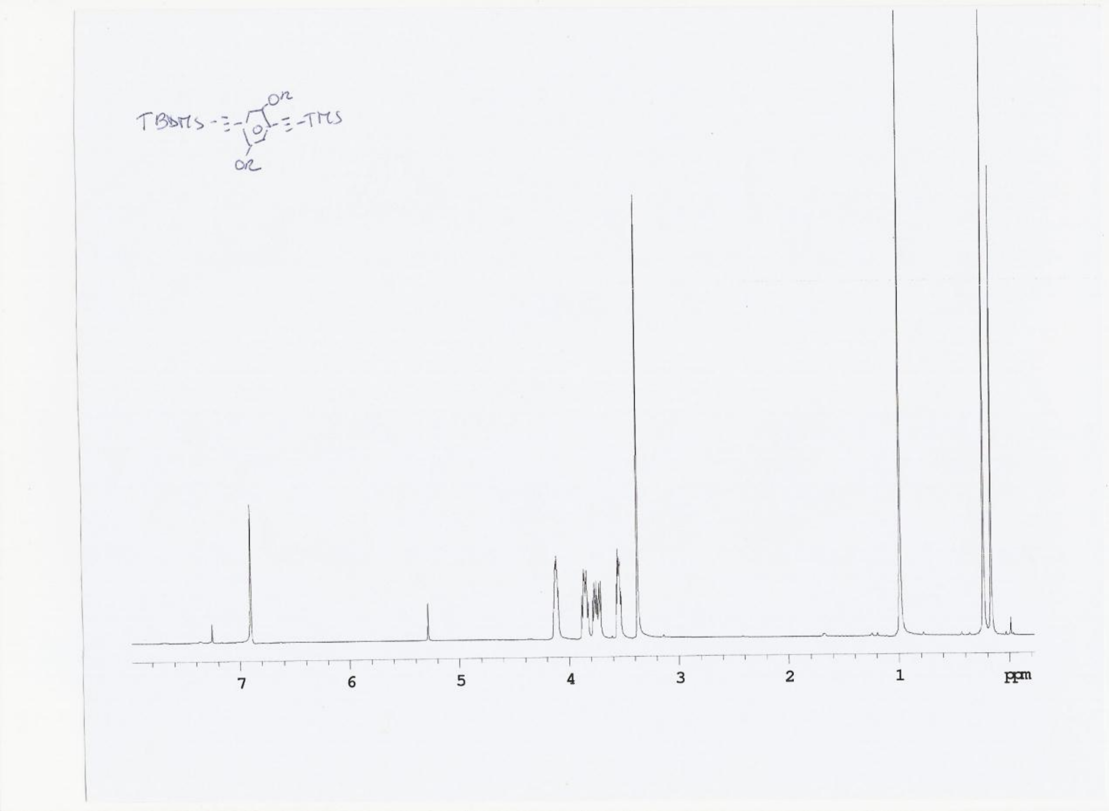

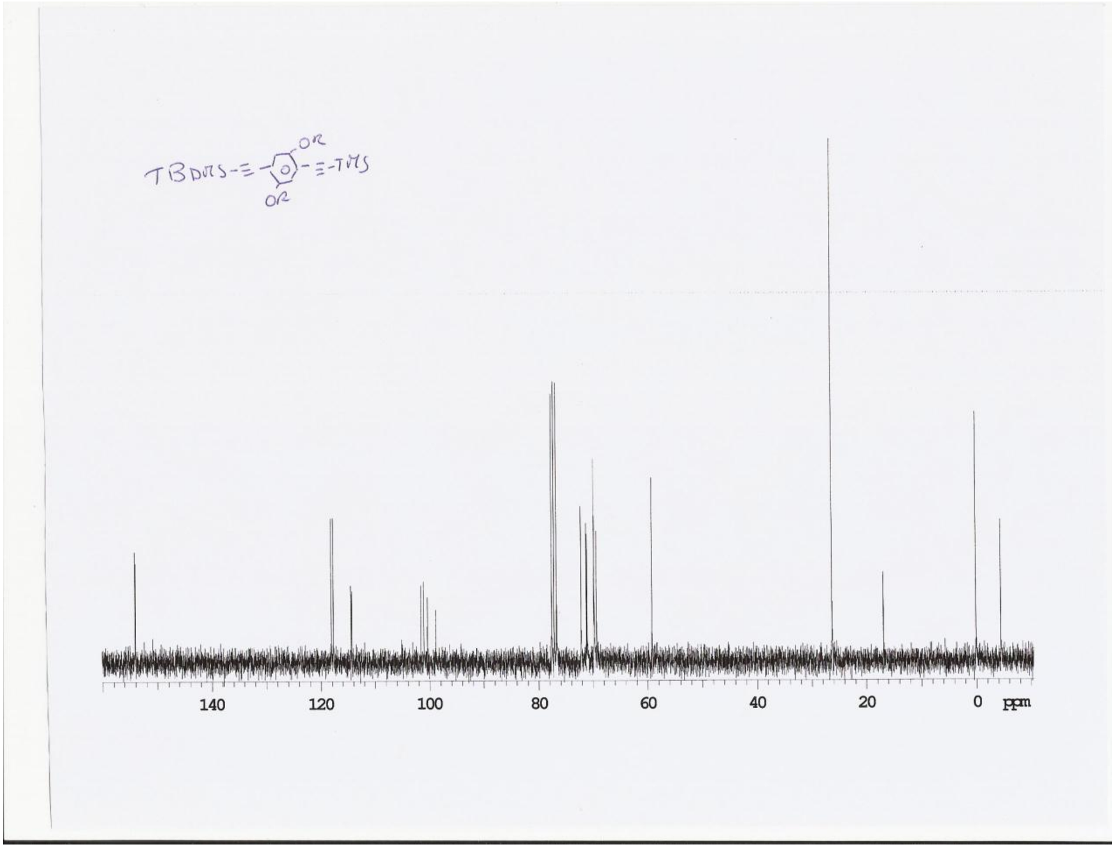

Compound **8** ^{1}H and ^{13}C NMR

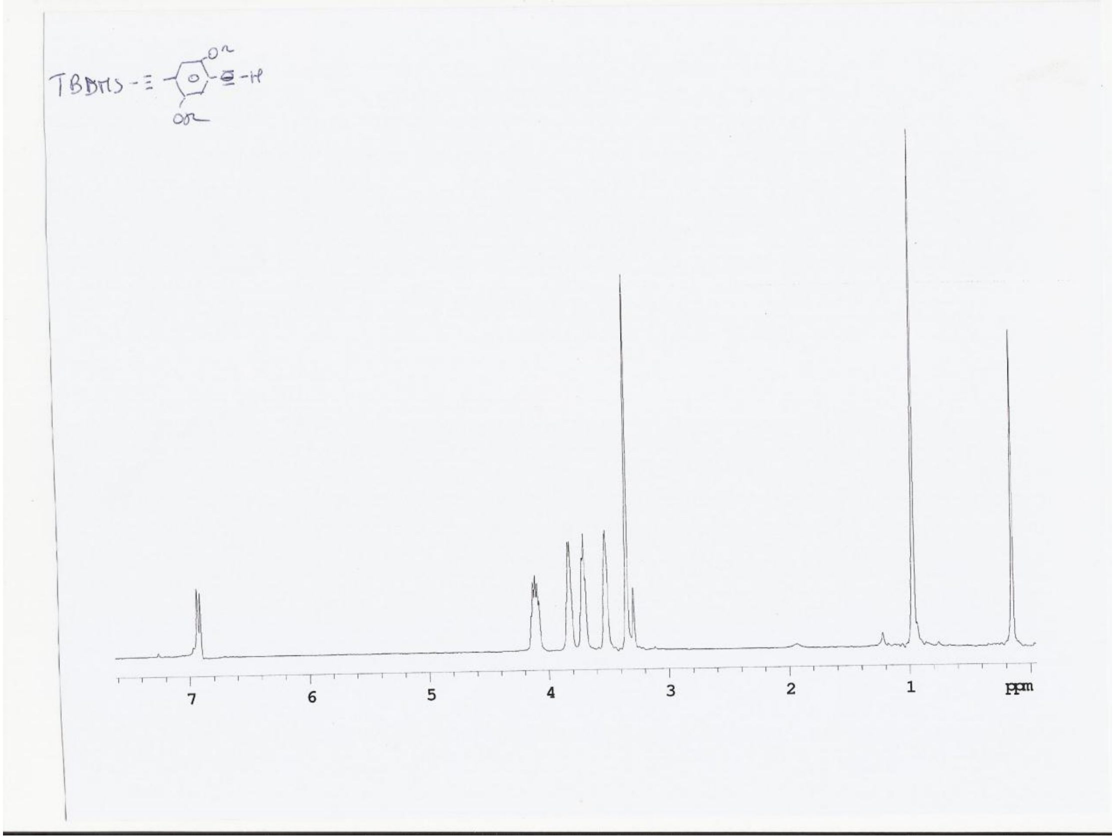

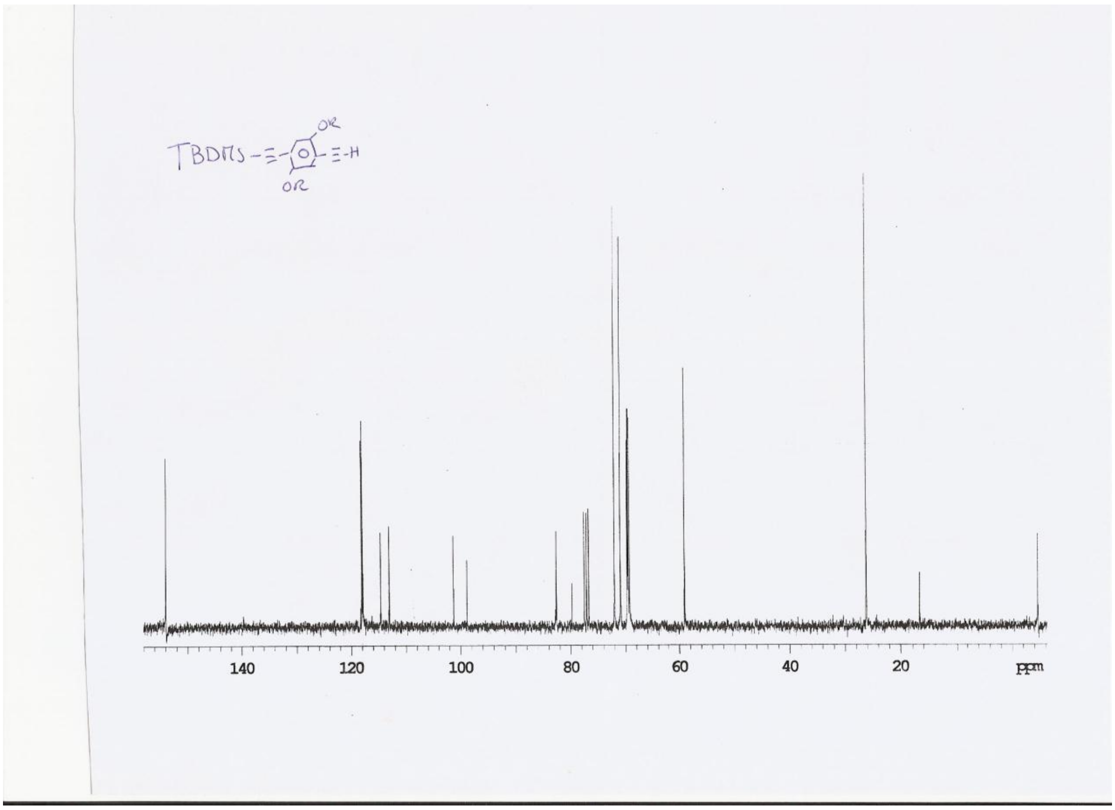

Compound **9** ^{1}H and ^{13}C NMR

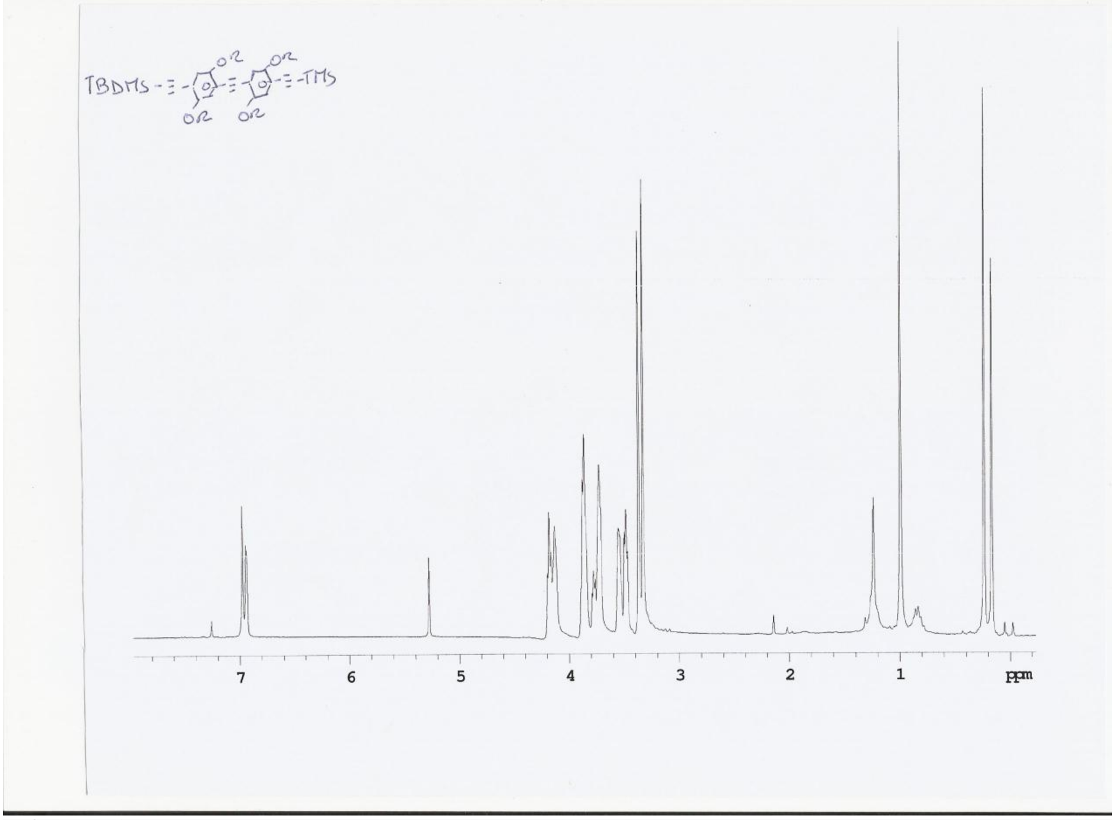

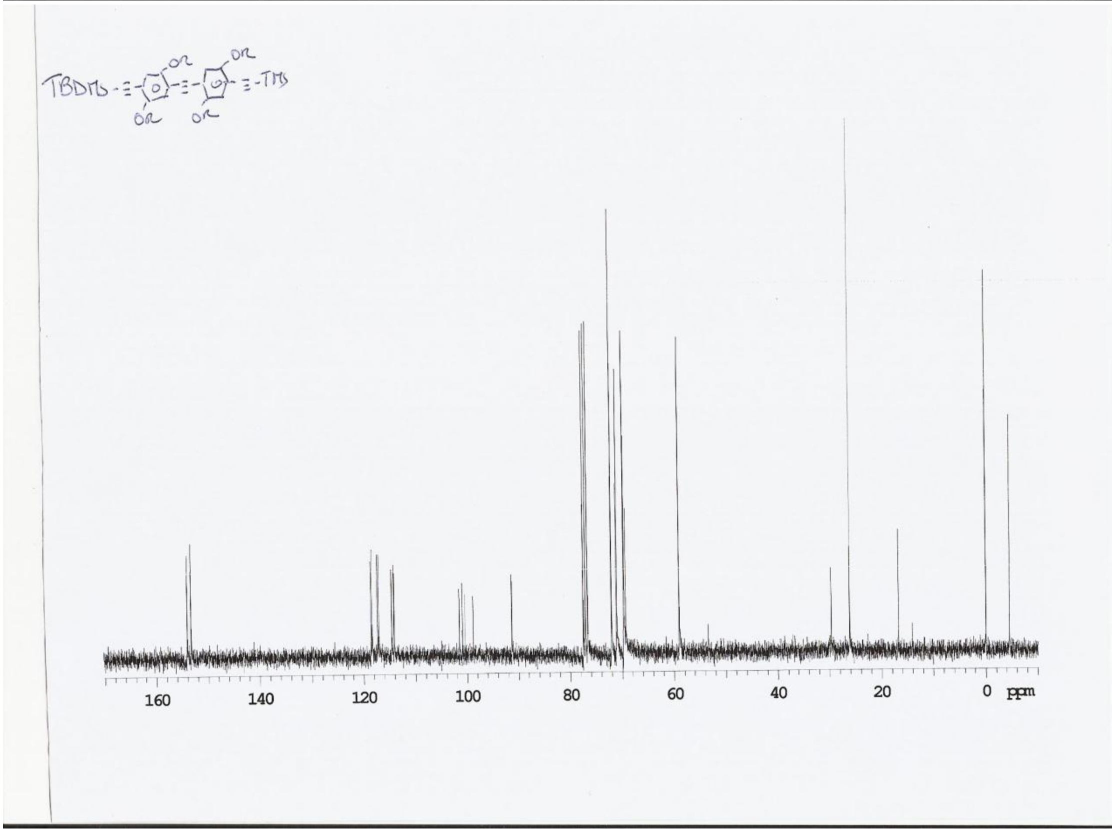

Compound **10** ^{1}H and ^{13}C NMR

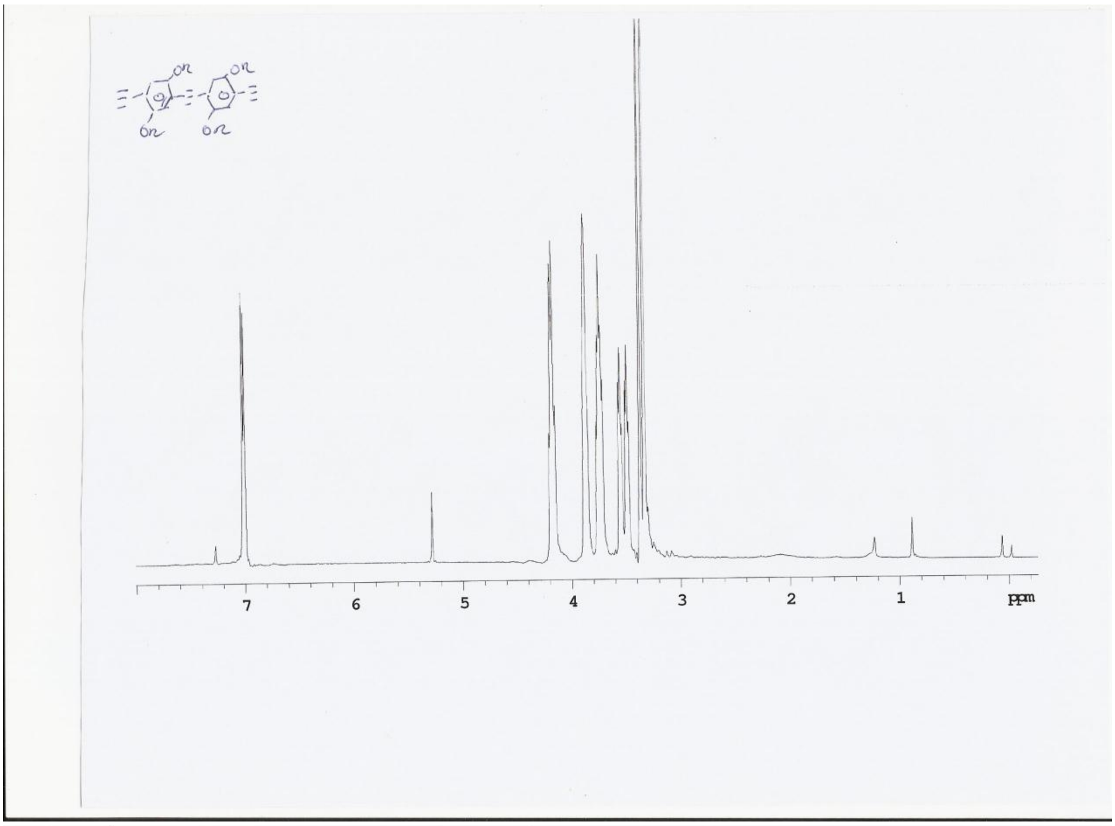

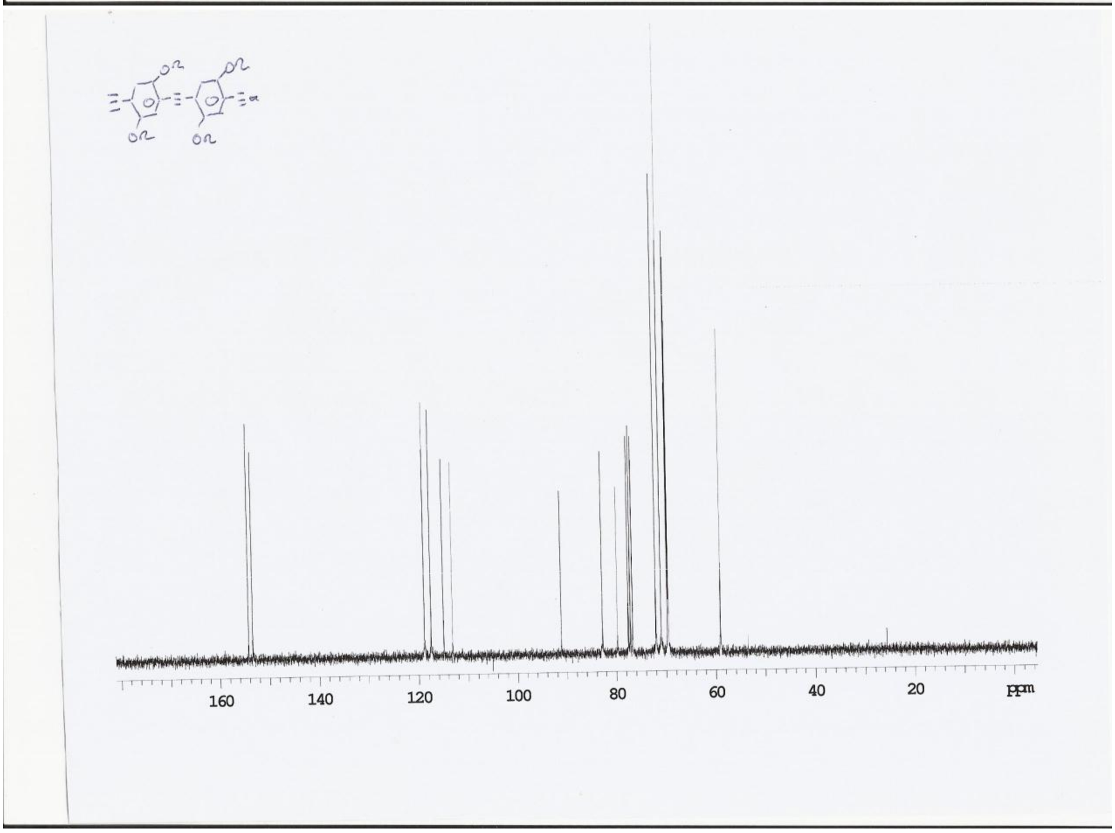

Compound **11** ^{1}H and ^{13}C NMR

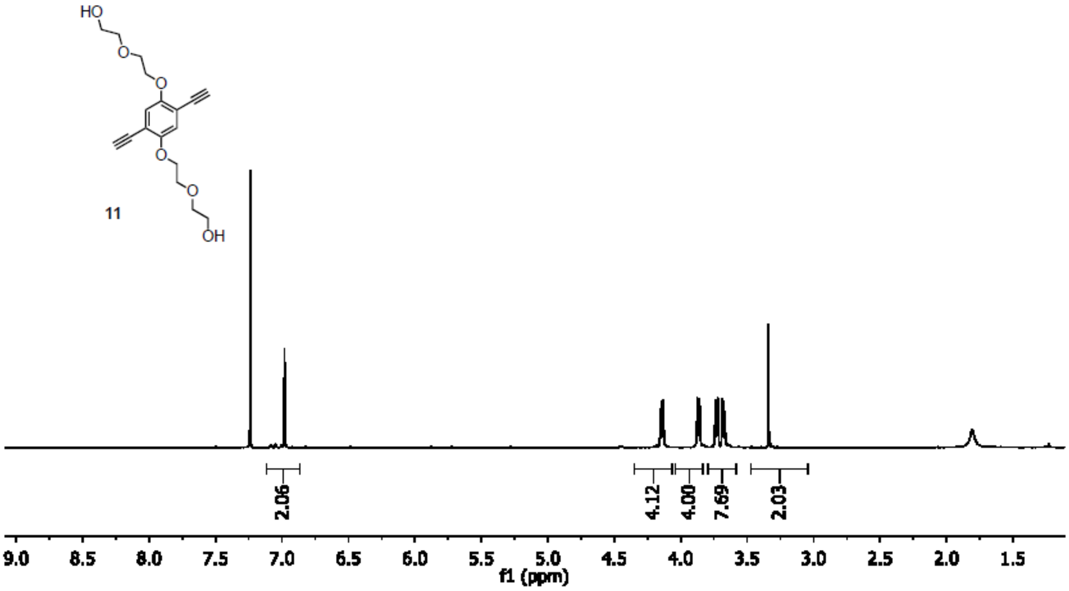

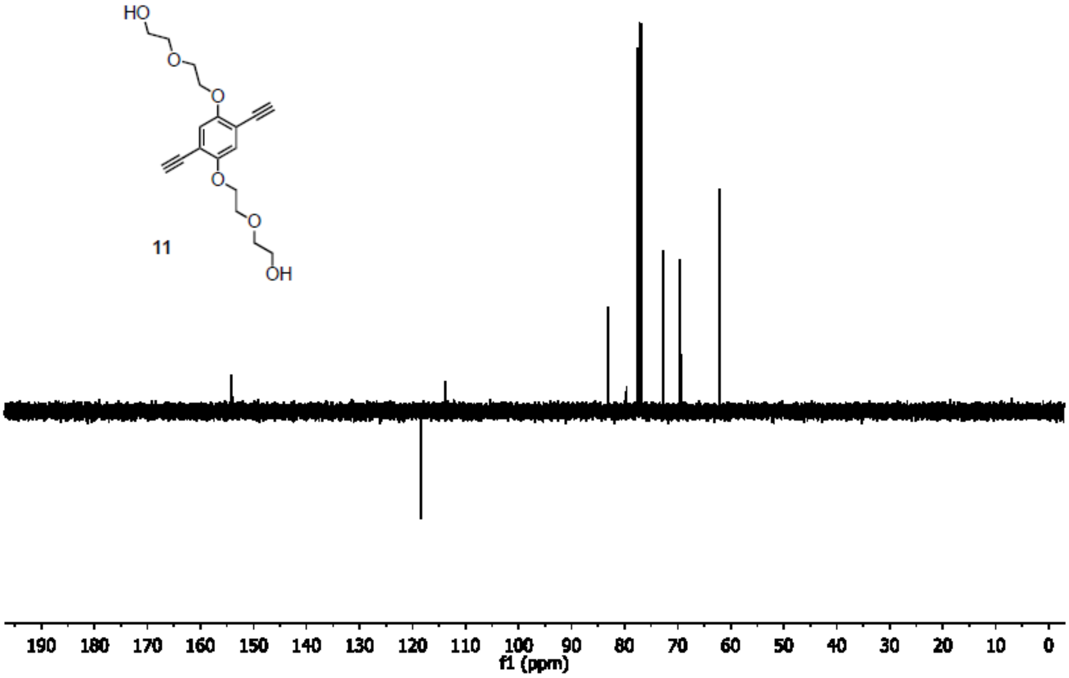

Compound **13** ^{1}H and ^{13}C NMR

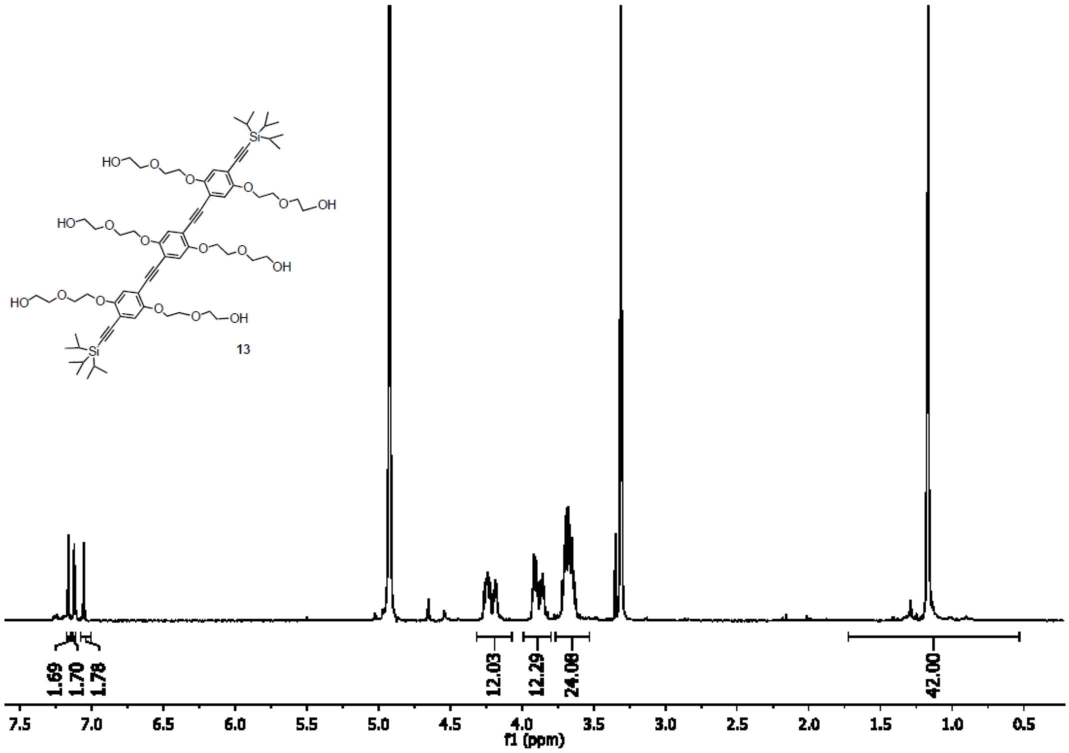

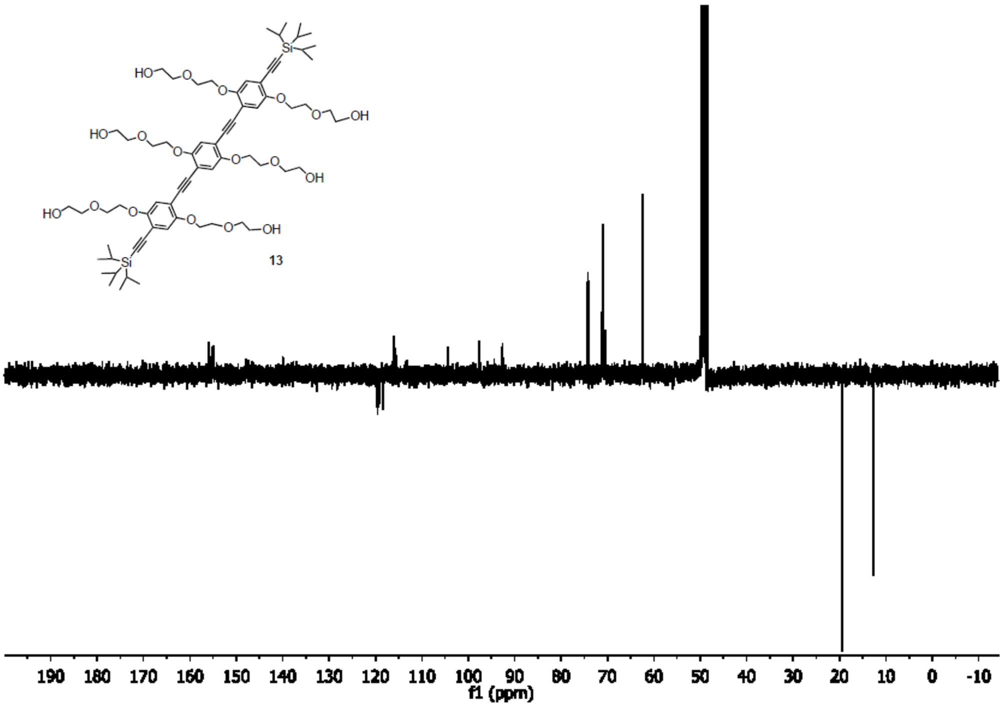

Compound **16** ^{1}H and ^{13}C NMR

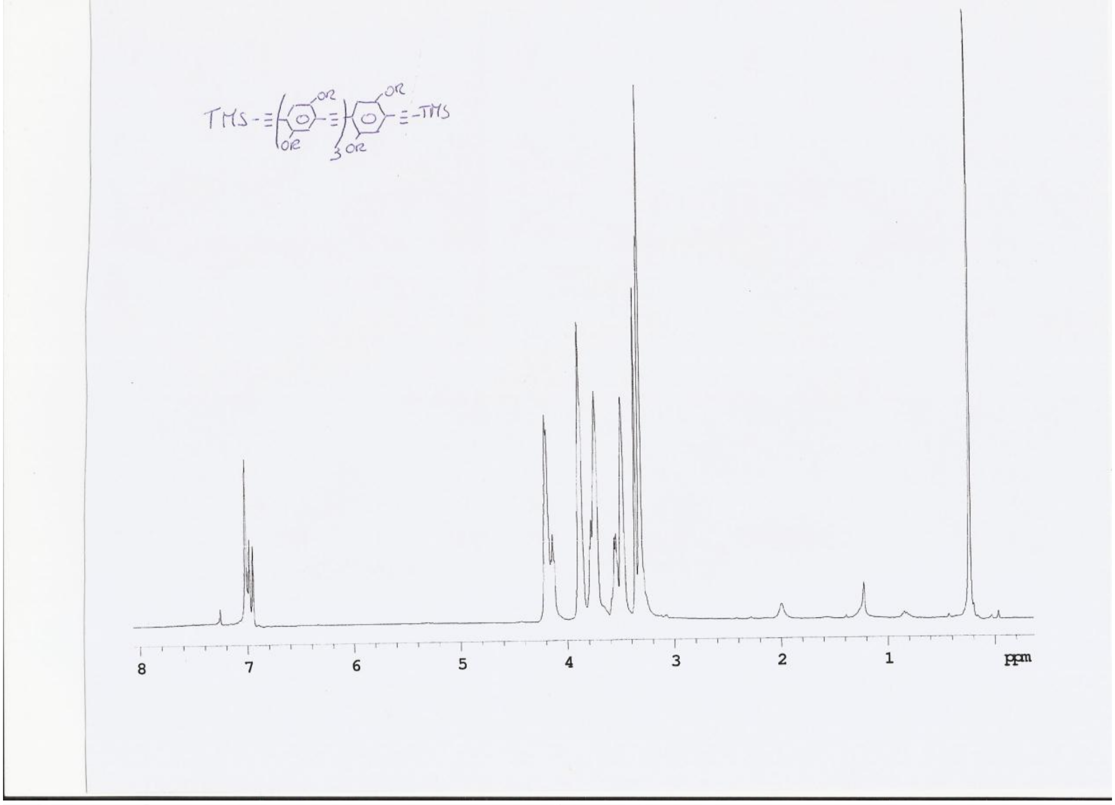

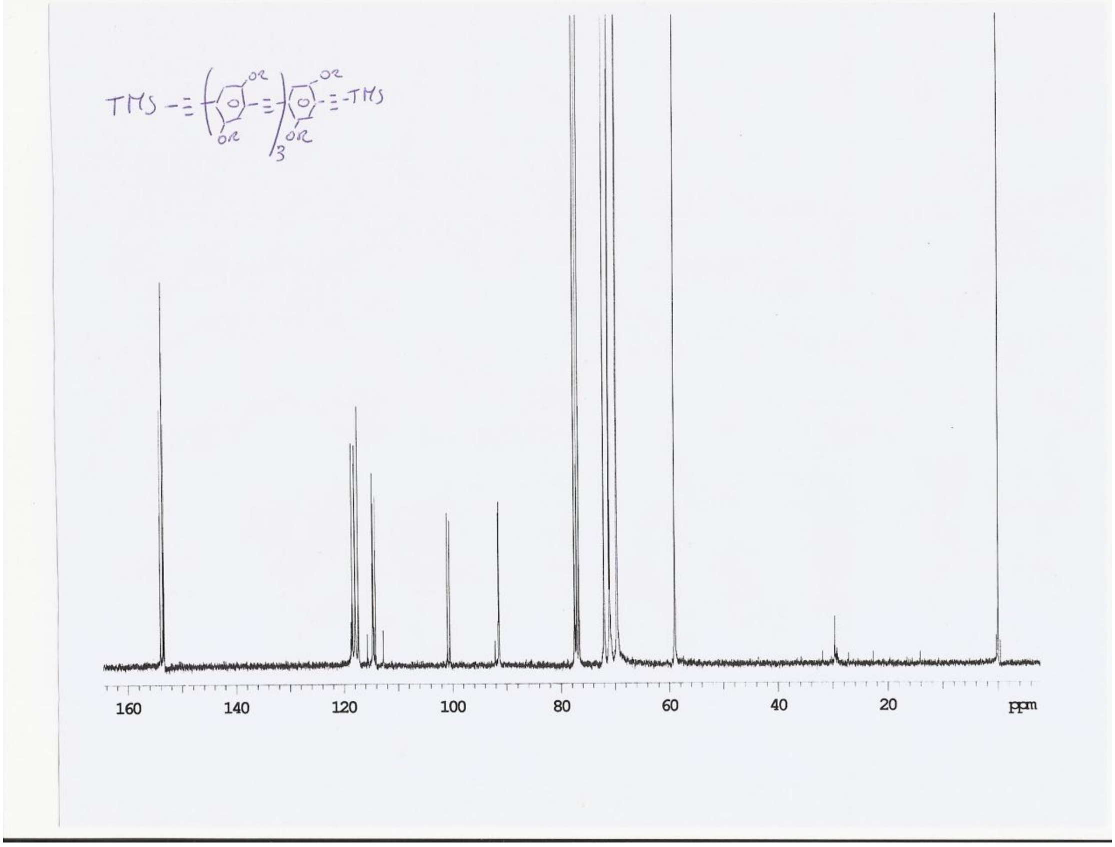

Compound **17** ^{1}H and ^{13}C NMR

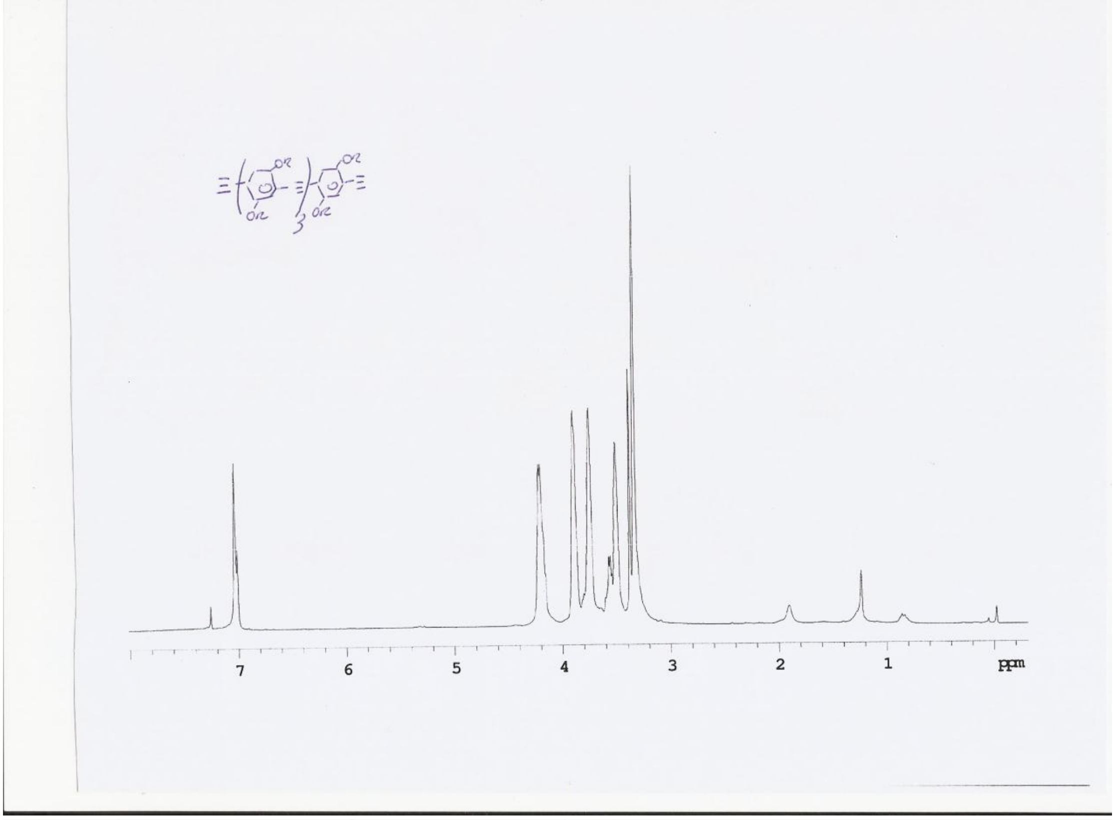

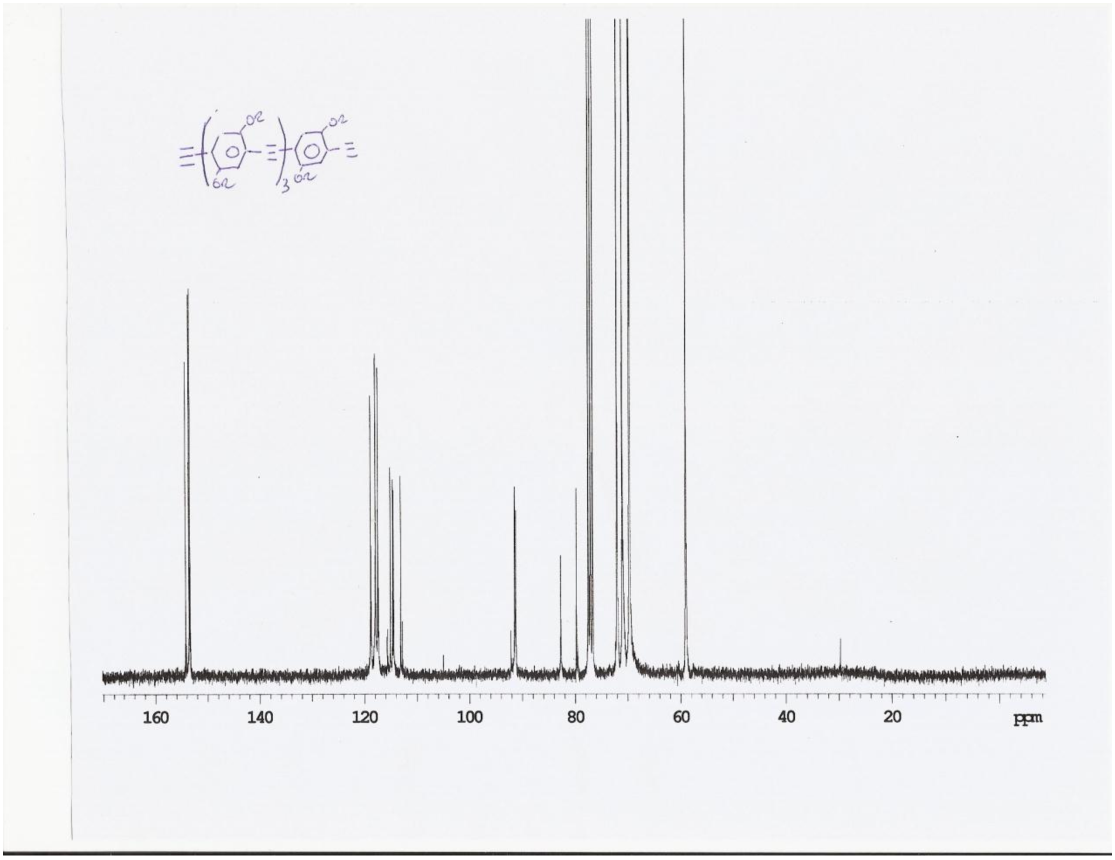

Compound **18** ^{1}H and ^{13}C NMR

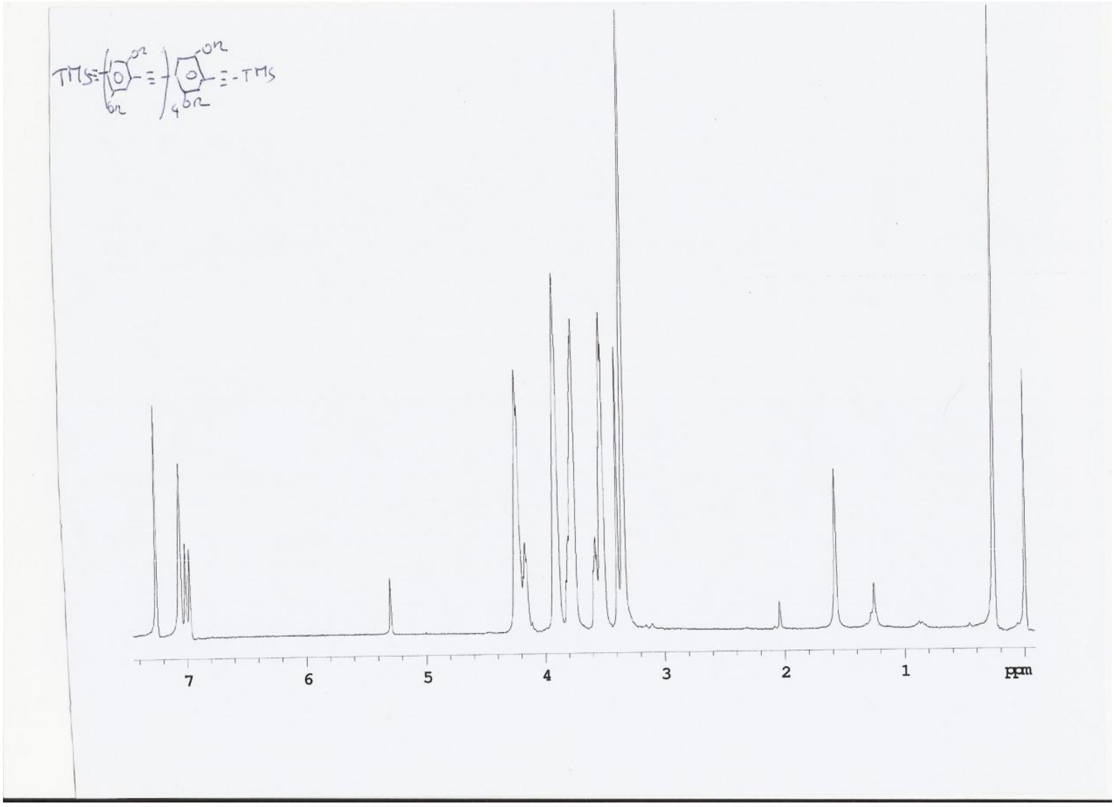

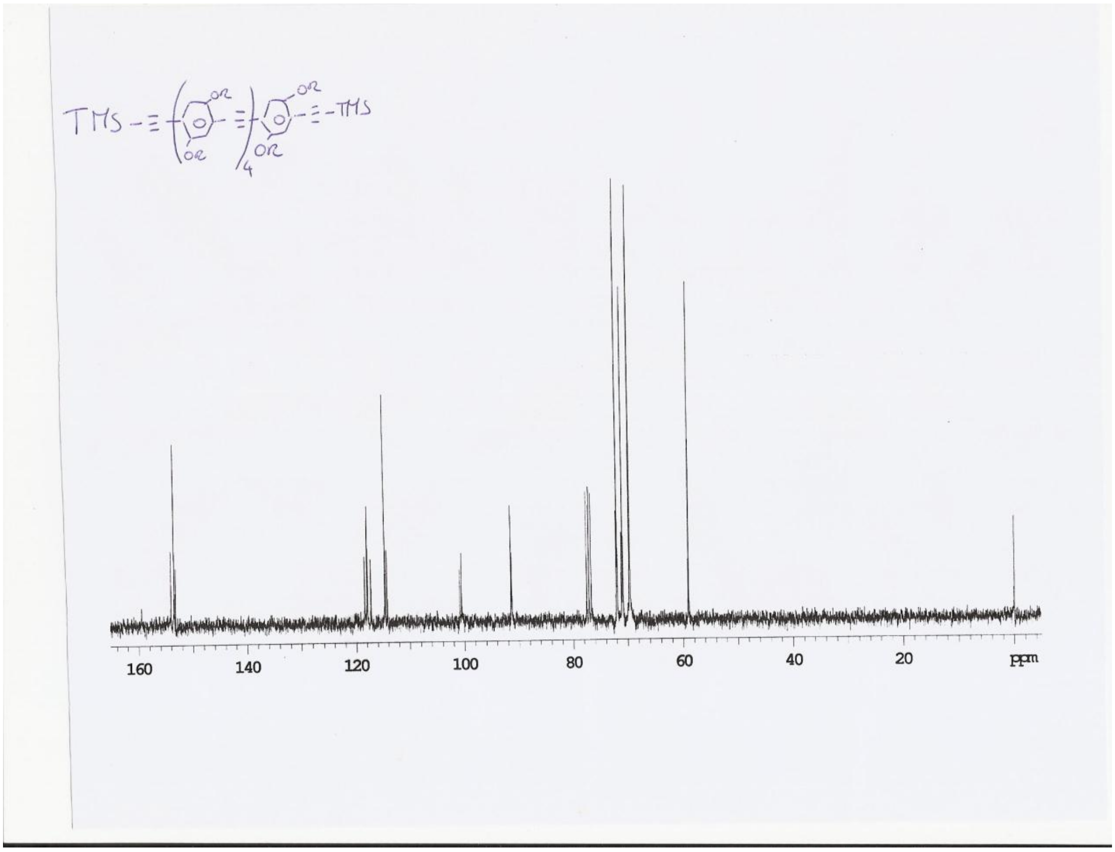

Compound **19** ^{1}H and ^{13}C NMR

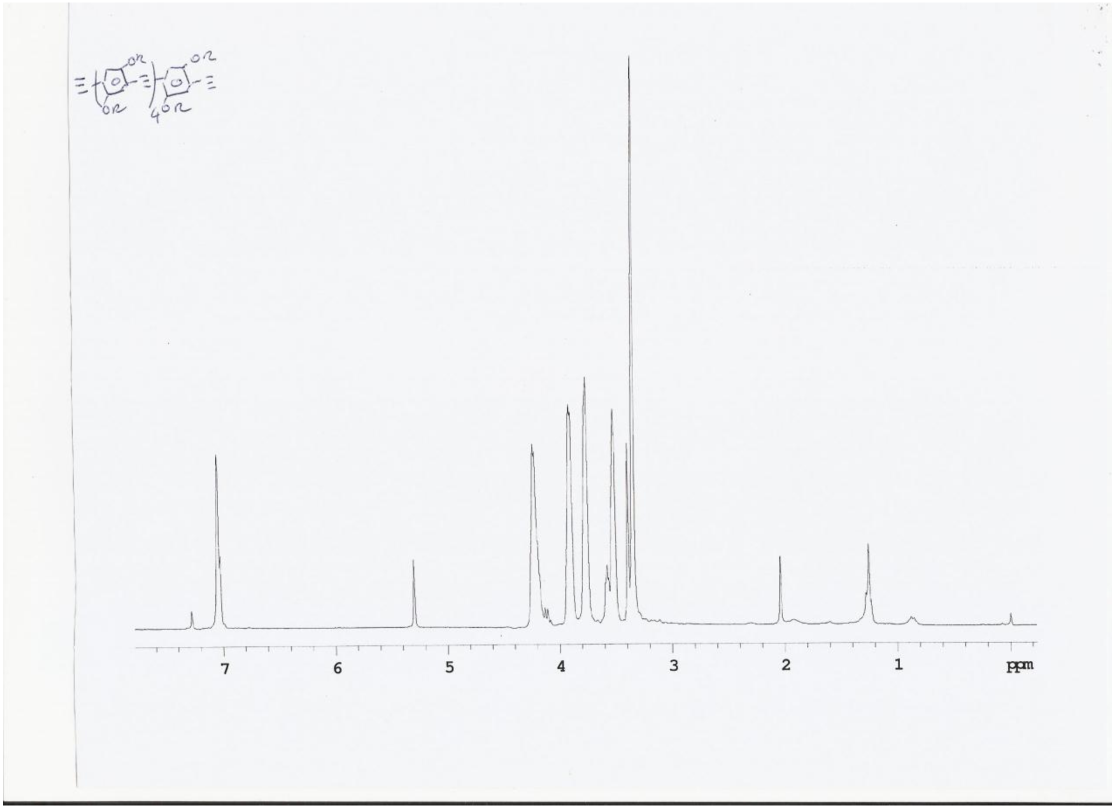

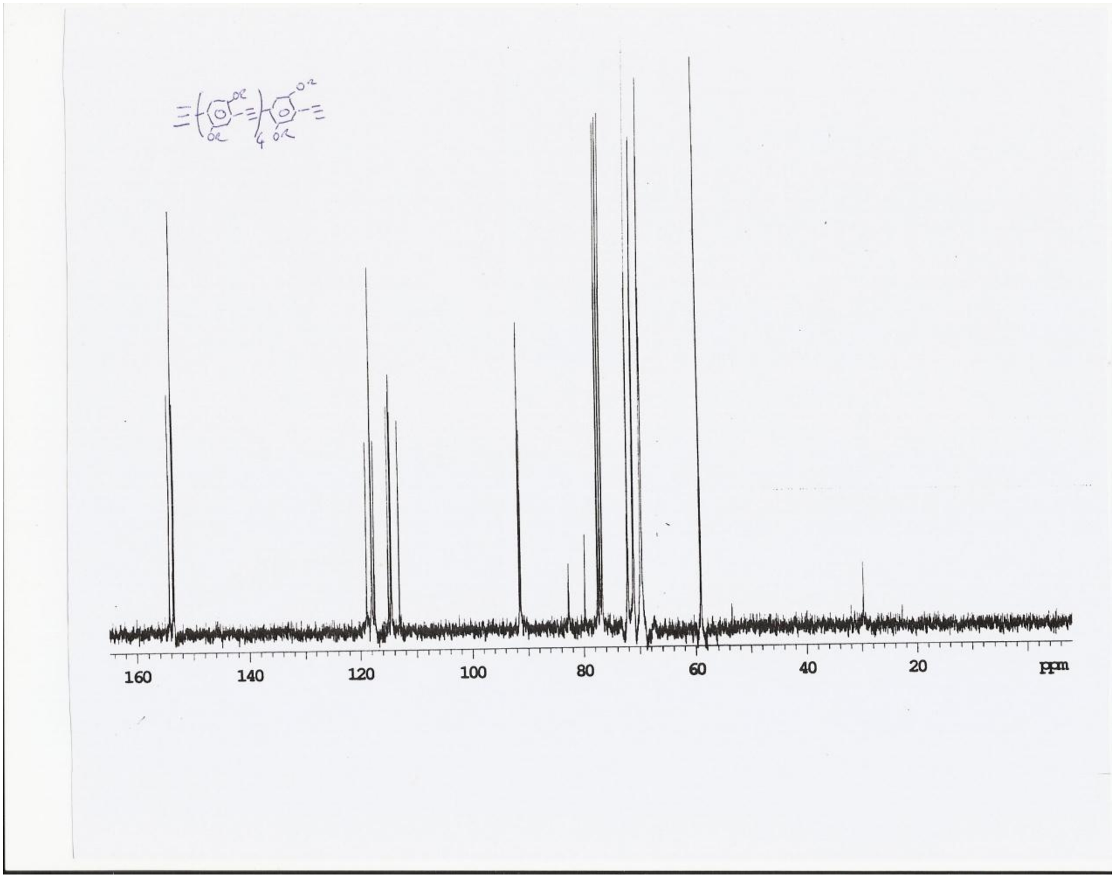

Compound **21** ^{1}H and ^{13}C NMR

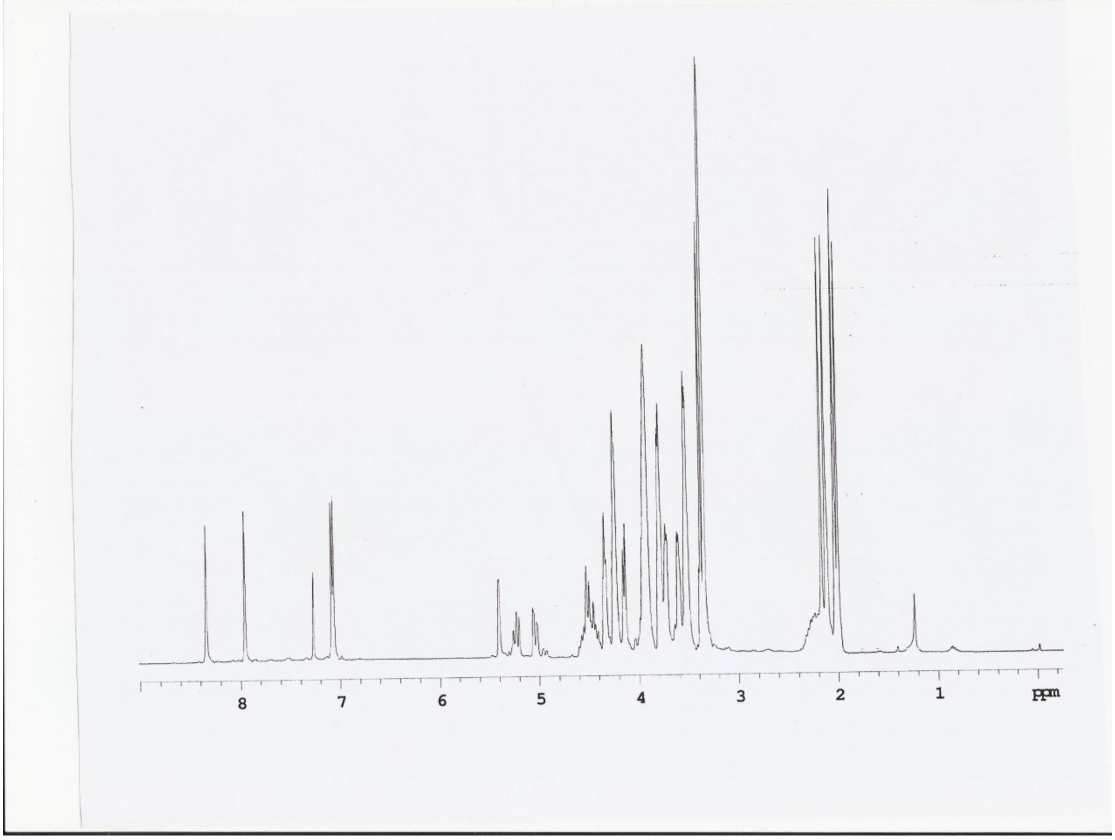

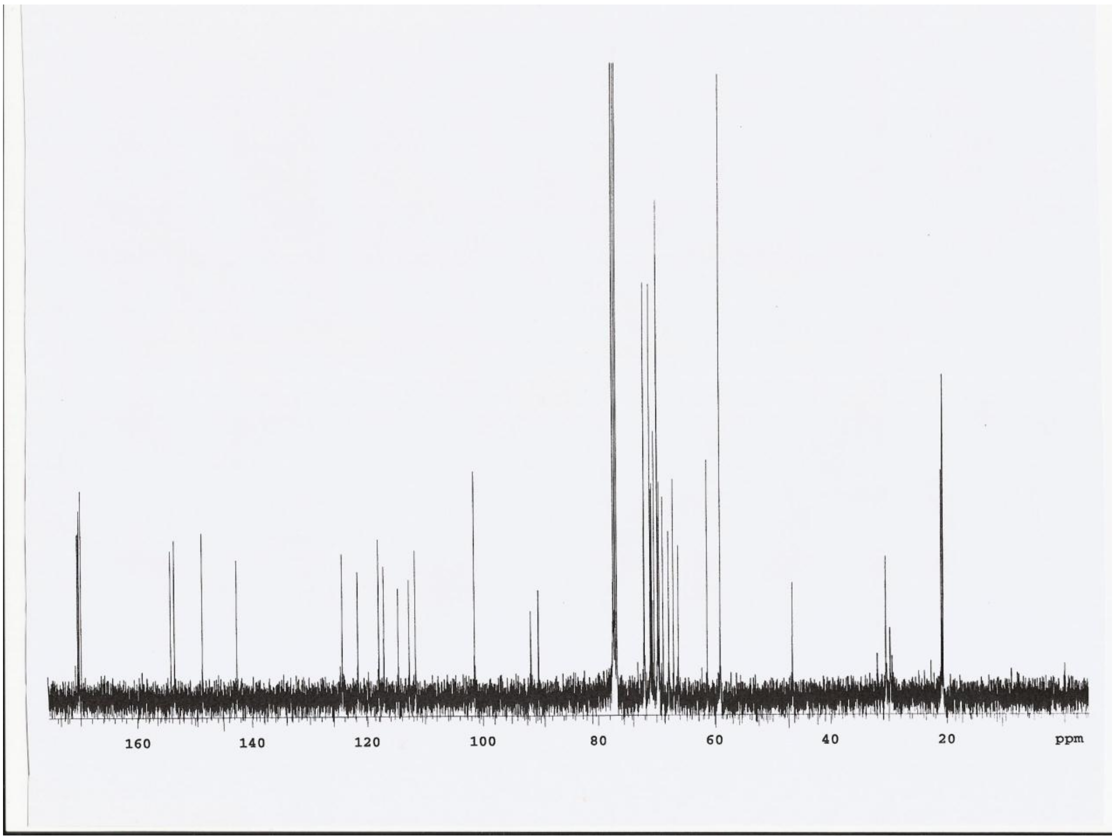

Compound **22** ^{1}H and ^{13}C NMR

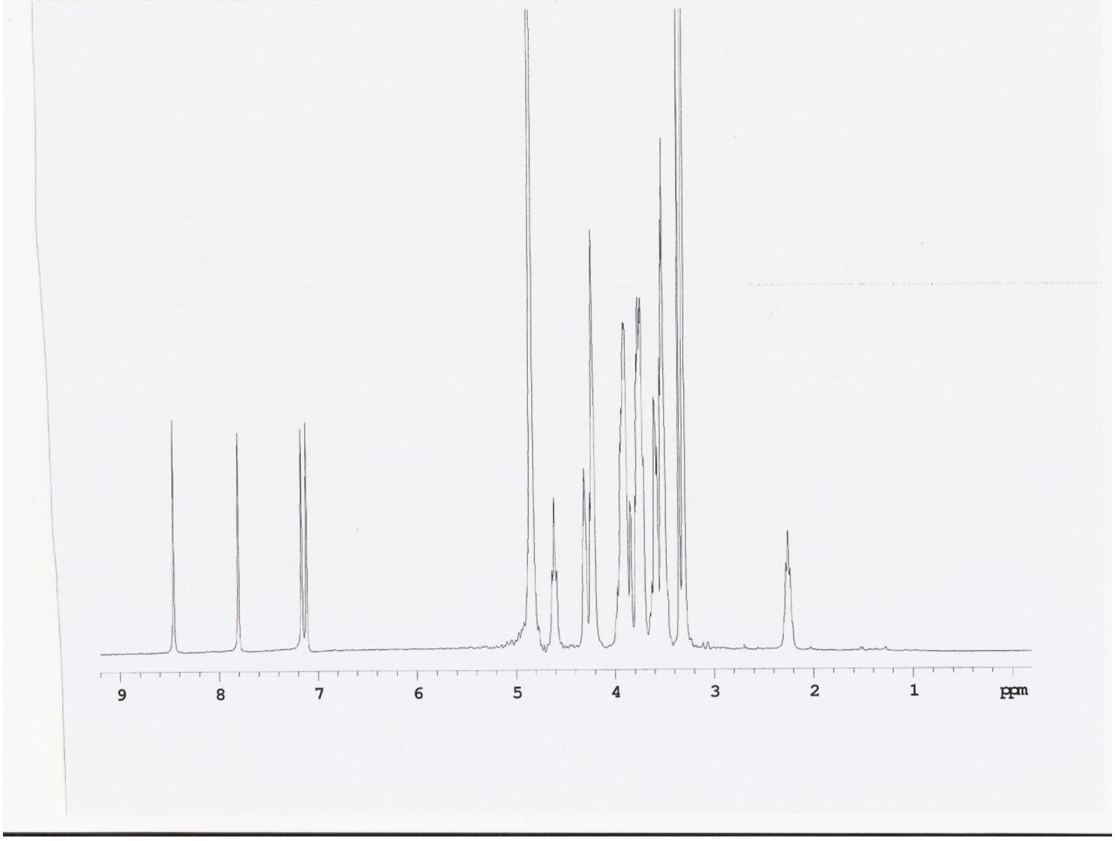

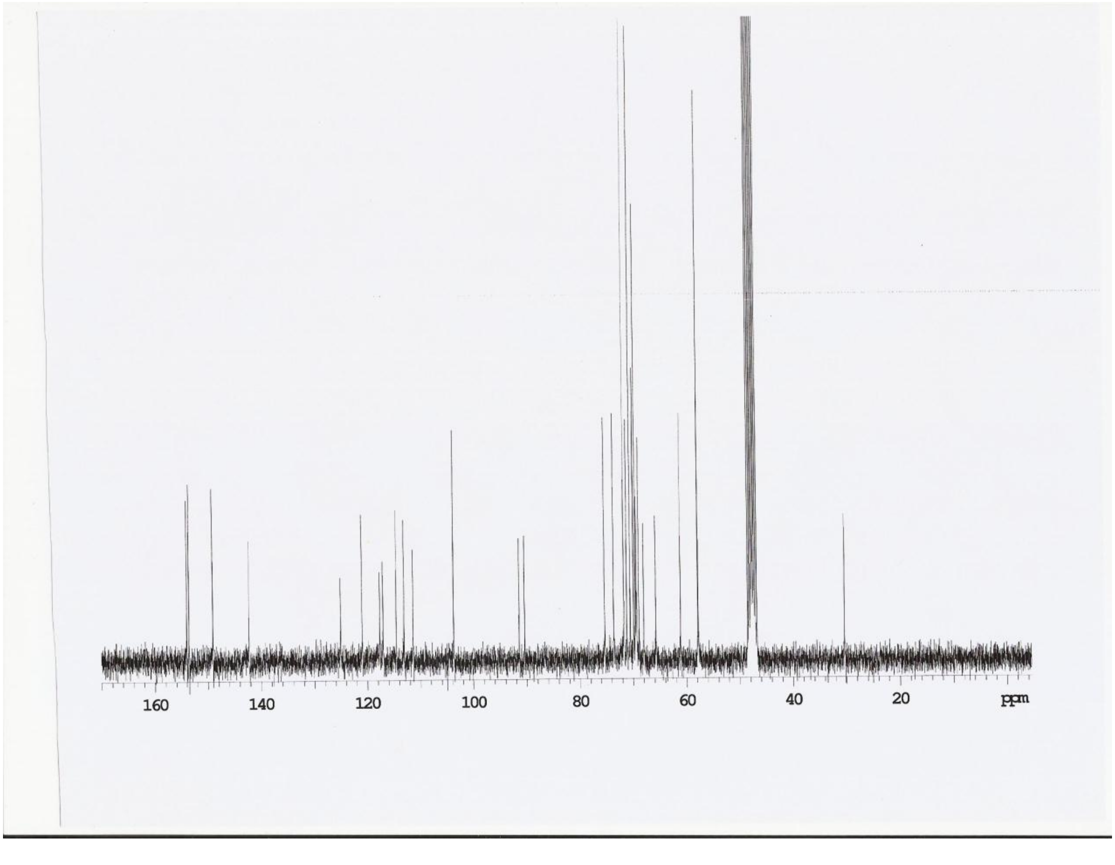

Compound **24** ^{1}H and ^{13}C NMR

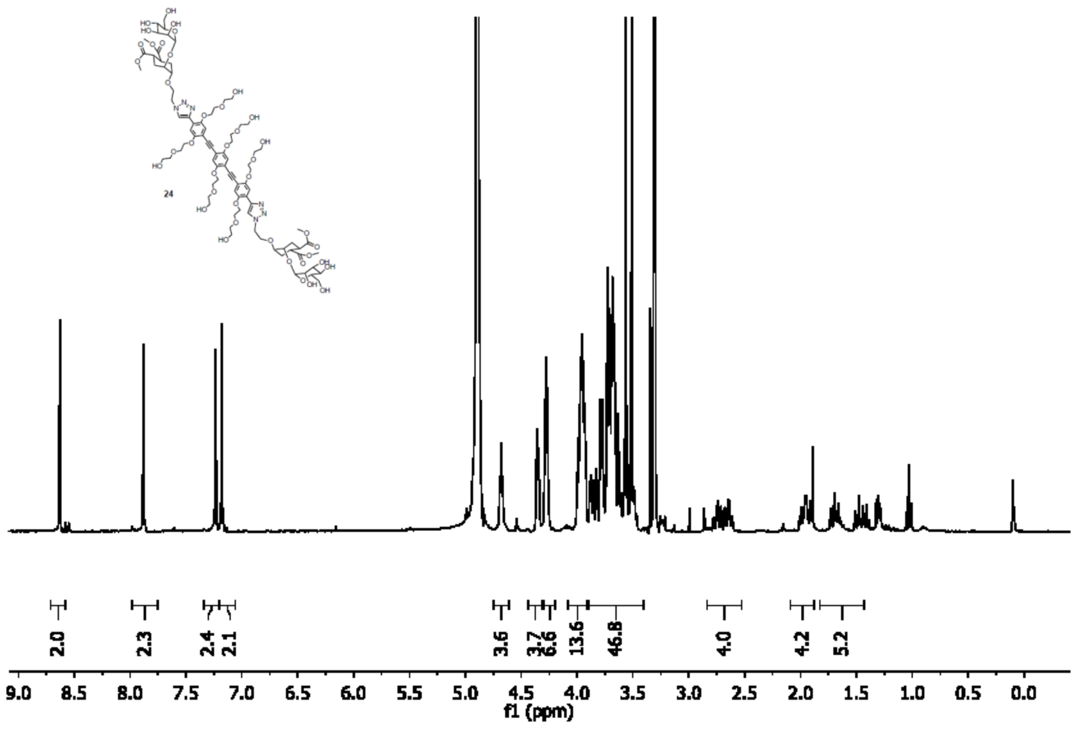

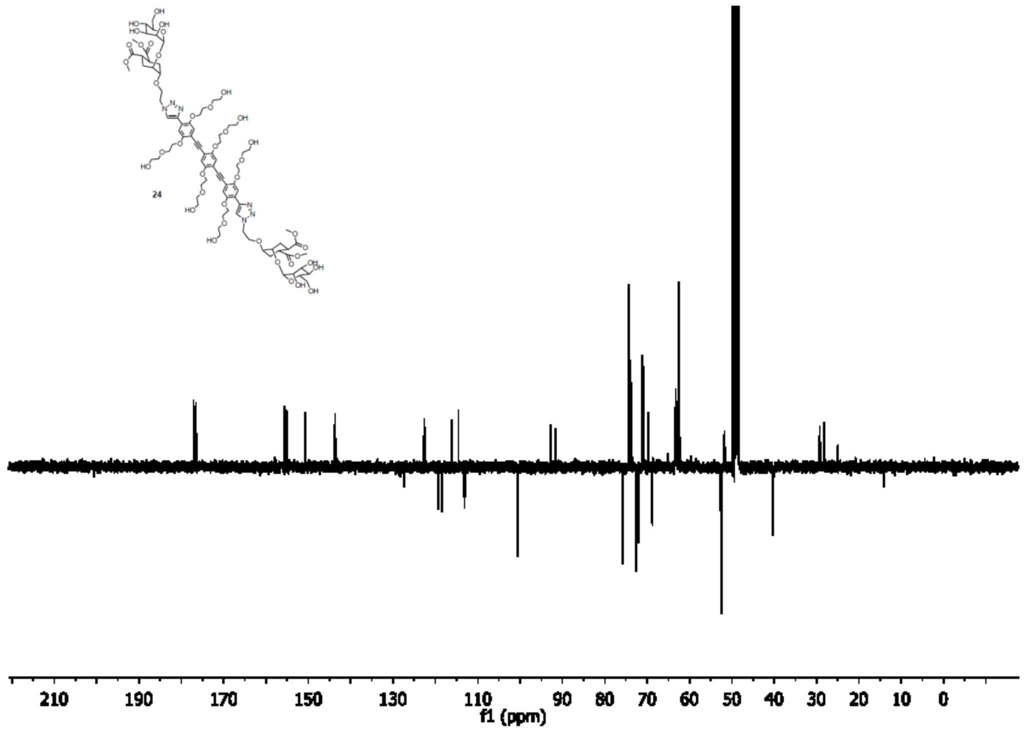

References

1. Sierra, C. A.; Lahti, P. M. *J. Phys. Chem. A* **2006,** *110,* 12081–12088. doi:10.1021/jp060543g
2. Li, J.; Kendig, C. E.; Nesterov, E. E. *J. Am. Chem. Soc.* **2007,** *129,* 15911–15918. doi:10.1021/ja0748027
3. Joosten, J. A. F.; Loimaranta, V.; Appeldoorn, C. C. M.; Haataja, S.; El Maate, F. A.; Liskamp, R. M. J.; Finne, J.; Pieters, R. J. *J. Med. Chem.* **2004,** *47,* 6499–6508. doi:10.1021/jm049476+
4. Goding, J. W. *J. Immunol. Methods* **1976,** *13,* 215–226. doi:10.1016/0022-1759(76)90068-5
5. Wu, Y.; Dong, Y.; Li, J.; Huang, X.; Cheng, Y.; Zhu, C. *Chem.–Asian J.* **2011,** *6,* 2725–2729. doi:10.1002/asia.201100534
